# Regulation of Phosphoribosyl-Linked Serine Ubiquitination by Deubiquitinases DupA and DupB

**DOI:** 10.1016/j.molcel.2019.10.019

**Published:** 2020-01-02

**Authors:** Donghyuk Shin, Rukmini Mukherjee, Yaobin Liu, Alexis Gonzalez, Florian Bonn, Yan Liu, Vladimir V. Rogov, Marcel Heinz, Alexandra Stolz, Gerhard Hummer, Volker Dötsch, Zhao-Qing Luo, Sagar Bhogaraju, Ivan Dikic

**Affiliations:** 1 Institute of Biochemistry II, Faculty of Medicine, Goethe University Frankfurt, Theodor-Stern-Kai 7, 60590 Frankfurt am Main, Germany; 2 Buchmann Institute for Molecular Life Sciences, Goethe University Frankfurt, Max-von-Laue-Str. 15, 60438 Frankfurt am Main, Germany; 3 Max Planck Institute of Biophysics, Max-von-Laue-Str. 3, 60438 Frankfurt am Main, Germany; 4 Purdue Institute of Immunology, Inflammation, and Infectious Diseases and Department of Biological Sciences, Purdue University, West Lafayette, IN 47907, USA; 5 Institute of Biophysical Chemistry and Center for Biomolecular Magnetic Resonance and Cluster of Excellence Macromolecular Complexes (CEF), Goethe University, Frankfurt, Germany; 6 Institute of Biophysics, Goethe University Frankfurt, 60438 Frankfurt am Main, Germany; 7 European Molecular Biology Laboratory, 71 Avenue des Martyrs, 38000 Grenoble, France

**Keywords:** phosphoribosyl serine ubiquitination, deubiquitinase, endoplasmic reticulum, *Legionella pneumophila*, deubiquitination, ADP-ribosylation, ER-fragmentation, phosphodiesterase, SdeA

## Abstract

The family of bacterial SidE enzymes catalyzes non-canonical phosphoribosyl-linked (PR) serine ubiquitination and promotes infectivity of *Legionella pneumophila*. Here, we describe identification of two bacterial effectors that reverse PR ubiquitination and are thus named deubiquitinases for PR ubiquitination (DUPs; DupA and DupB). Structural analyses revealed that DupA and SidE ubiquitin ligases harbor a highly homologous catalytic phosphodiesterase (PDE) domain. However, unlike SidE ubiquitin ligases, DupA displays increased affinity to PR-ubiquitinated substrates, which allows DupA to cleave PR ubiquitin from substrates. Interfering with DupA-ubiquitin binding switches its activity toward SidE-type ligase. Given the high affinity of DupA to PR-ubiquitinated substrates, we exploited a catalytically inactive DupA mutant to trap and identify more than 180 PR-ubiquitinated host proteins in *Legionella*-infected cells. Proteins involved in endoplasmic reticulum (ER) fragmentation and membrane recruitment to *Legionella*-containing vacuoles (LCV) emerged as major SidE targets. The global map of PR-ubiquitinated substrates provides critical insights into host-pathogen interactions during *Legionella* infection.

## Highlights

• Phosphoribosyl serine ubiquitination can be reverted by DupA and DupB
• DUP specifically binds to and cleaves PR-ubiquitin from serine on substrates
• Catalytically inactive DupA mutants can capture PR-ubiquitinated proteins
• PR ubiquitination on multiple ER structural proteins causes ER fragmentation

## Graphical Abstract

**Figure:**
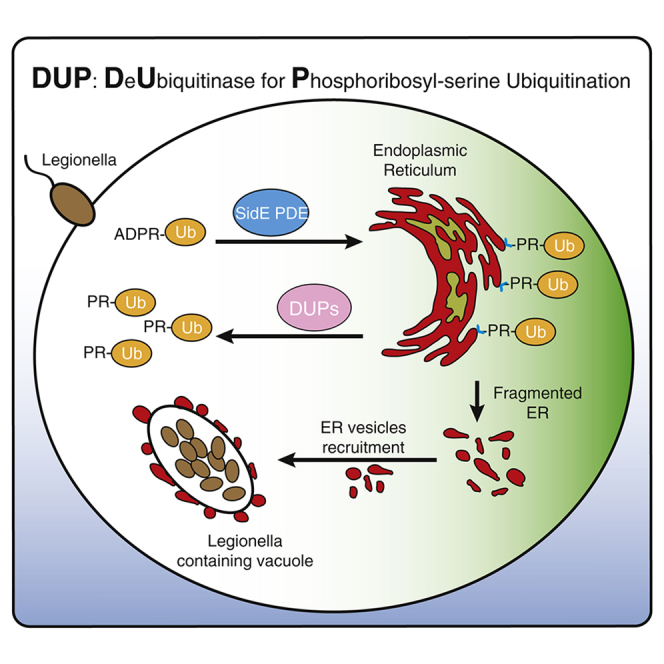

## Teaser

Shin et al. show that phosphoribosyl serine ubiquitination can be reversed by two deubiquitinases (DupA and DupB) from *Legionella.* Two DUPs specifically cleave PR-ubiquitin from serine on substrates. Catalytically inactive DupA-based proteomics approach reveals PR-ubiquitinated proteins and their roles in endoplasmic reticulum (ER) remodeling.

## Introduction

Ubiquitination is one of the most versatile post-translational modifications, controlling a wide variety of cellular processes (Hochstrasser, 2009). In most cases, the carboxy terminus of ubiquitin (Ub) is covalently linked to the ε-amino (a primary amine) group of one or more lysines on substrates. Subsequent additions of further Ub moieties create polymers of Ub, which have diverse structures and functions (Yau and Rape, 2016). These Ub structures can be recognized by specific receptors that contain Ub-binding domains (UBDs) that can result in the delivery of ubiquitinated substrate to the proteasome for degradation or to selective autophagy pathways to changes in protein function and/or cellular localization (Dikic, 2017). The mechanism underlying the ubiquitination process is well established. It involves a cascade of three enzymes: E1-Ub activating enzyme, E2-Ub conjugating enzyme, and E3-Ub ligase. Specific enzymes called deubiquitinases (DUBs) cleave off Ub from substrates and regulate the abundance of ubiquitinated proteins (Clague et al., 2019).

Given the importance of Ub signaling, a considerable number of pathogens utilize virulence factors that modulate Ub and autophagy systems to promote pathogenicity (Grohmann et al., 2018, Hicks and Galán, 2013, Llosa et al., 2009, Maculins et al., 2016, Qiu and Luo, 2017). This is clearly demonstrated by *Legionella pneumophila*, a Gram-negative bacterium that causes Legionnaires’ disease and possesses the largest number of documented bacterial effectors among intracellular bacterial pathogens (Burstein et al., 2016). For example, the *Legionella* effectors LegU1 and LeuAU13 serve as F-box-containing E3 ligases that interact with host Cul1-Skp1 and ubiquitinate BAT3, a host chaperone protein (Ensminger and Isberg, 2010). Another effector is LubX, a RING and U-box type E3 ligase, which, in conjunction with the host E2 enzymes UbcH5a or UbcH5c, ubiquitinates host Clk1 kinase (Kubori et al., 2008, Quaile et al., 2015). More recently, *Legionella pneumophila* was also shown to utilize a non-canonical type of ubiquitination through the action of enzymes belonging to the SidE family of effectors (SdeA, SdeB, SdeC, and SidE) (Bhogaraju et al., 2016, Qiu et al., 2016). This NAD-dependent modification involves the conjugation of Ub via a phosphoribosyl (PR) moiety to serine residues of host substrates (Bhogaraju et al., 2016, Qiu et al., 2016). SidE-type enzymes contain two intrinsic enzymatic domains: the mono ADP-ribosyl transferase (mART) domain that utilizes NAD to transfer ADP-ribose (ADPR) on Arg42 of Ub and the phosphodiesterase (PDE) domain that cleaves ADPR-Ub to PR-Ub and conjugates PR-Ub to substrate serines (Akturk et al., 2018, Dong et al., 2018, Kalayil et al., 2018, Wang et al., 2018). Among the known PR-ubiquitinated substrates are several ER-associated Rab GTPases and reticulon 4 (Rtn4). Upon infection, *Legionella pneumophila* regulates dynamics of membranes to create a *Legionella*-containing vacuole (LCV) where they can reside and avoid the host defense system. PR ubiquitination has been shown to impair GTP-loading and GTP-hydrolysis activity of Rab GTPases (Qiu et al., 2016) and tubular ER rearrangements and potential fragmentation of ER in order to promote proliferation of bacteria in the LCV (Kotewicz et al., 2017). Recent evidence also shows a role of SidE family effectors in regulating mTORC1 activity through PR ubiquitination of Rag GTPases on the lysosome (De Leon et al., 2017). Moreover, the *Legionella* effector SidJ has been proposed to act as a deubiquitinase for both conventional and PR-linked ubiquitination (Qiu et al., 2017); however, recent findings indicate that SidJ acts as a glutamylase that inhibits SidE enzymes by targeting the catalytic site of the ART domains (Bhogaraju et al., 2019, Black et al., 2019, Gan et al., 2019).

Despite these findings, critical questions related to the spectrum of PR-ubiquitinated substrates and the associated functional consequences as well as the dynamics of PR ubiquitination remain to be explored. In this study, we address these issues by identifying two bacterial effectors encoding deubiquitinases for PR-linked ubiquitination (DUPs), which counteract the activity of SidE ligases by removing PR-ubiquitin from substrate serines. We also provide biophysical and structural explanations for their specificity toward PR-ubiquitinated substrates. Moreover, based on their strong binding affinity to PR-ubiquitinated substrates, we have engineered an inactive DupA variant that acts as a trapping mutant for endogenously PR-ubiquitinated substrates in *Legionella*-infected cells. This approach enabled us to identify multiple classes of PR-ubiquitinated substrates. We also show that PR ubiquitination is required for ER fragmentation and ER recruitment to LCV upon *Legionella* infection. Collectively, these findings provide invaluable insights into *Legionella*-mediated PR ubiquitination of host proteins and shed light on the functional relevance of this modification upon infection.

## Results

### Identification of DUPs

**Figure 1:**
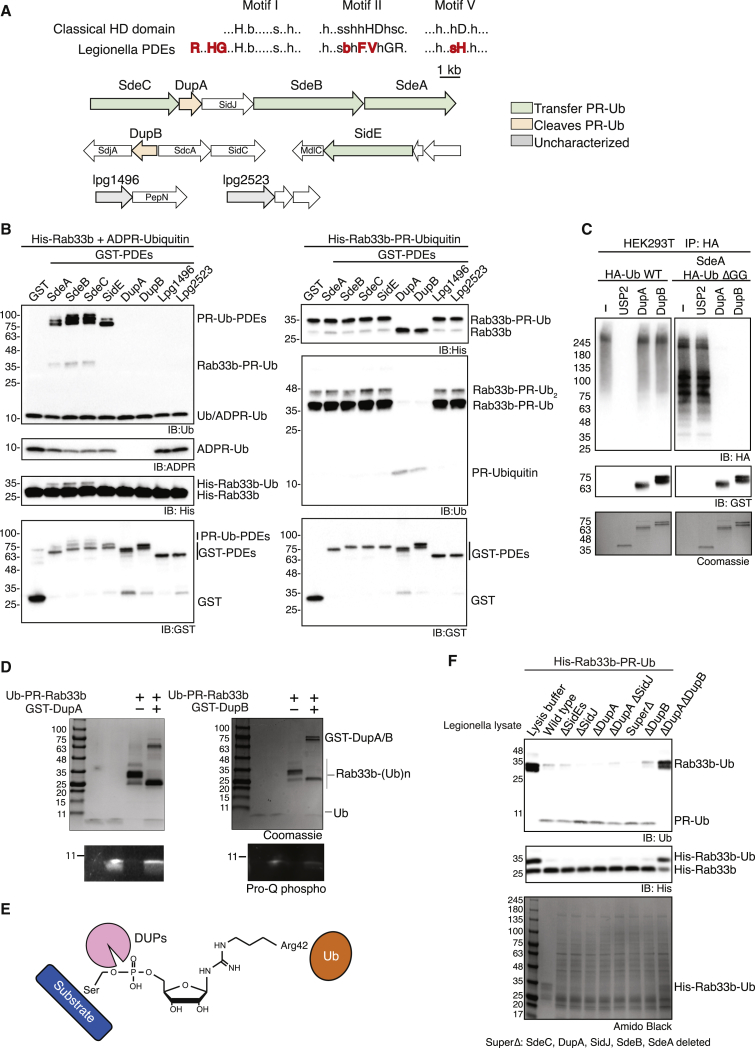
Identification of Novel Deubiquitinating Enzymes Specific for Phosphoribosyl-Linked Serine Ubiquitination (A) Comparison between conserved amino acids of a classical HD domain and *Legionella* PDEs. Unique amino acids for PDEs are highlighted in red, and a pattern used for finding other *Legionella* PDEs is presented. Single letter abbreviations for amino acids are as follows: b, bulky; c, charged; h, hydrophobic; s, small; x, any residue; D, Asp; F, Phe; G, Gly; H, His; L, Leu; R, Arg; T, Thr; V, Val (upper). Gene loci of eight PDE-containing proteins from *Legionella* and their neighboring genes (lower). (B) PR ubiquitination (left) and deubiquitination (right) assays with eight PDEs. (C) Deubiquitination assay of USP2 and DupA/B on cell lysates. Ub without the two C-terminal glycine residues was used to monitor PR ubiquitination. (D) Phospho-staining of cleaved Ub species from PR-ubiquitinated Rab33b after DupA/B treatment. (E) Schematic chemical representation of phospho-ribose linkage between Ub and substrates. (F) PR-deubiquitination assay of *Legionella* lysates. PR-ubiquitinated Rab33b was incubated with *Legionella* lysates as indicated.

The transfer of PR-Ub to substrate serine residues by SidEs is mediated by their PDE domains (Akturk et al., 2018, Bhogaraju et al., 2016, Kalayil et al., 2018), which resemble classical HD (histidine and aspartate) domains (Aravind and Koonin, 1998, Morar et al., 2015). Based on sequence similarity, we identified four additional SidE-like PDE-containing *Legionella* proteins (Lpg1496, Lpg2523, Lpg2154 (or LaiE), and Lpg2509 (LaiF or SdeD); Figures 1A and S8). Sequence alignment revealed that the catalytic residues of the SdeA PDE domain (E340, H277, and H407) (Akturk et al., 2018, Kalayil et al., 2018) are highly conserved in all eight PDE-containing *Legionella* proteins. Despite this high conservation, incubation of ADPR-Ub with the newly identified PDE-containing proteins did not result in autoubiquitination and/or Rab33b ubiquitination (Figure 1B, left). Instead, LaiE and LaiF (Luo and Isberg, 2004) PDE domains processed the ADPR-Ub but did not transfer the PR-Ub to the substrate *in vitro* (Figure 1B, left). Importantly, these PDE domains cleaved PR-ubiquitinated Rab33b (Rab33b-PR-Ub) *in vitro* (Figure 1B, right) and multiple PR-ubiquitinated substrates in cells (Figure 1C). As such, we renamed these *Legionella* effectors as DUPs: DupA/LaiE and DupB/LaiF. Moreover, both DupA and DupB specifically cleaved PR-ubiquitinated substrates but not canonical lysine-linked ubiquitination substrates (Figure 1C). Further biochemical analyses also revealed that the released Ub species were stained by phosphoprotein staining solution (Figure 1D), indicating that DupA and DupB cleaved the bond between PR-Ub and the substrate serine residue. This finding was further confirmed by mass spectrometry analysis (Figures S1A and 1E). To examine whether there are other proteins cleaving PR-Ub from serine, we generated a *Legionella* strain without DUPs and mixed lysates with PR-ubiquitinated Rab33b. Depletion of both DUPs, but not SidJ, which has been previously suggested to serve as a PR-ubiquitin specific deubiquitinase, failed to hydrolyze PR-ubiquitin from Rab33b (Figure 1F). Collectively, our data establish a new class of deubiquitinases specific for PR ubiquitination that includes DupA and DupB.

### Structural and Functional Analyses of DupA PDE Domain

**Figure 2:**
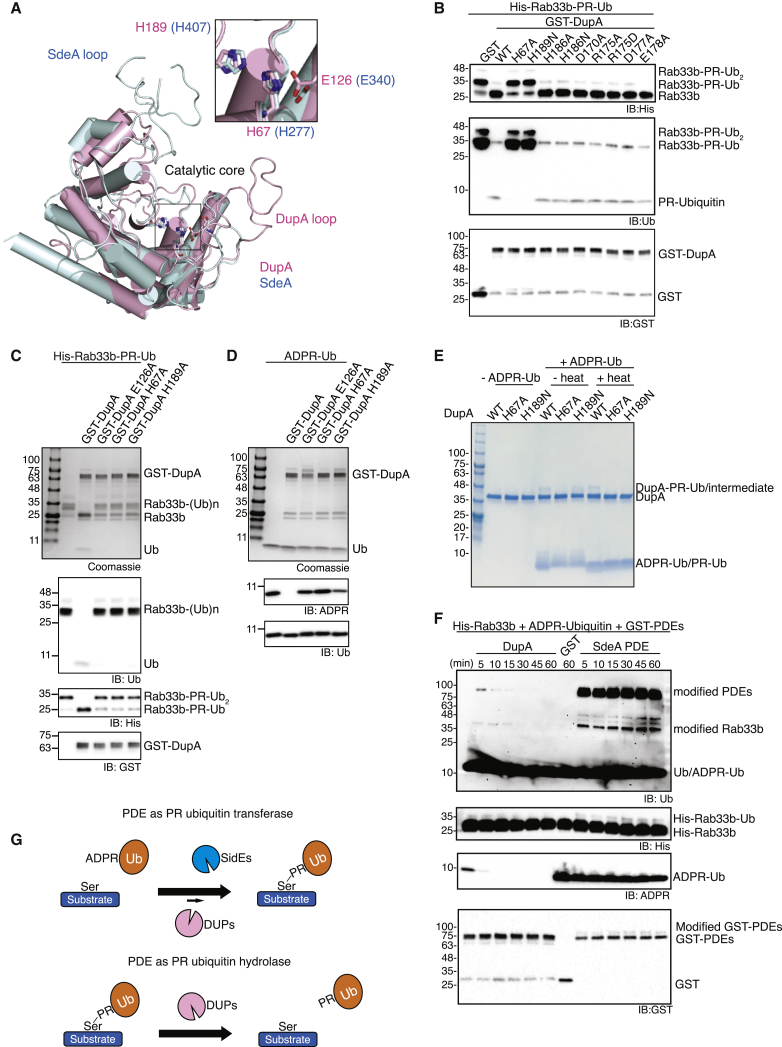
Catalytic Mechanism of DupA (A) X-ray crystal structure of DupA is superimposed with structure of SdeA PDE (PDB: 6G0C). Subset shows catalytic site of two PDE domains, and catalytic residues (H67, H189, and E126) are shown as stick model. (B) Identification of new catalytic residues on DupA. Putative catalytic residues were mutated as indicated and subjected to deubiquitination assay. (C and D) Deubiquitination assay of WT and catalytically inactive DupA mutants on PR-ubiquitinated Rab33b and ADPR-Ub, respectively. (E) Histidine intermediate assay of DupA. DupA WT and mutants were incubated with ADPR-Ub for 5 min and analyzed on SDS-PAGE. (F) Time course PR ubiquitination assay of DupA and SdeA PDE. His-Rab33b and ADPR-Ub were incubated with DupA or SdeA PDE for indicated time points and analyzed. (G) A schematic of PR ubiquitination and deubiquitination. See also Figure S1.

To elucidate the molecular mechanism of DUPs, we determined the crystal structure of DupA_4-345_ (PDB: 6RYB, Figure 2A). The overall structure of DupA resembled the PDE domains of the SidE family ligases SdeA (Kalayil et al., 2018) and SidE (Wang et al., 2018) as well as the PDE domain of DupB (Akturk et al., 2018). The three catalytic residues from SdeA and DupB PDE domains were also highly conserved in the core of DupA (H67, E126, and H189), while several loop regions differed (Figure 2A). Varying the length of the corresponding SdeA loop, deleting the loop regions from both SdeA and DupA PDE domains, or swapping the loop region did not affect the function of either PDE domains (Figures S1B–S1D). In contrast, mutation of the three conserved catalytic residues from both DupA and DupB resulted in impaired cleavage activity (Figures 2B–2D, S1E, and S1F). Moreover, after 5 min of reaction, we detected a labile and heat-sensitive His-Ub intermediate on the DupA His-189-Asn mutant, while wild-type (WT) DupA displayed heat-stable auto-PR ubiquitination , which was further cleaved by DupA at later time points (Figures 2E, 2F, and S1G). This suggests that, similar to the SdeA PDE domain, DupA utilizes a histidine-based intermediate reaction to catalyze the hydrolysis of ADPR-Ub (Kalayil et al., 2018). Thus, the PDE domains of SidEs and DupA/B may share the same catalytic residues to mediate opposite reactions: PR-Ub transfer to substrates and removal of PR-Ub from substrates (deubiquitination), respectively. We next monitored both catalytic reactions over an extended time (Figures 2F and S1G). Upon incubation of ADPR-Ub and Rab33b substrate with DupA, small amounts of DupA autoubiquitination and Rab33b PR ubiquitination were detected at the very beginning of the reaction (5–15 min), which declined at later time points (30–60 min). This indicates that the DupA PDE domain, a strong deubiquitinase (or hydrolase), also has weak transferase activity *in vitro* involving the hydrolysis of ADPR-Ub and the transfer of PR-Ub to a substrate.

### DupA Interactions with Ub Define Its Catalytic Activity

**Figure 3:**
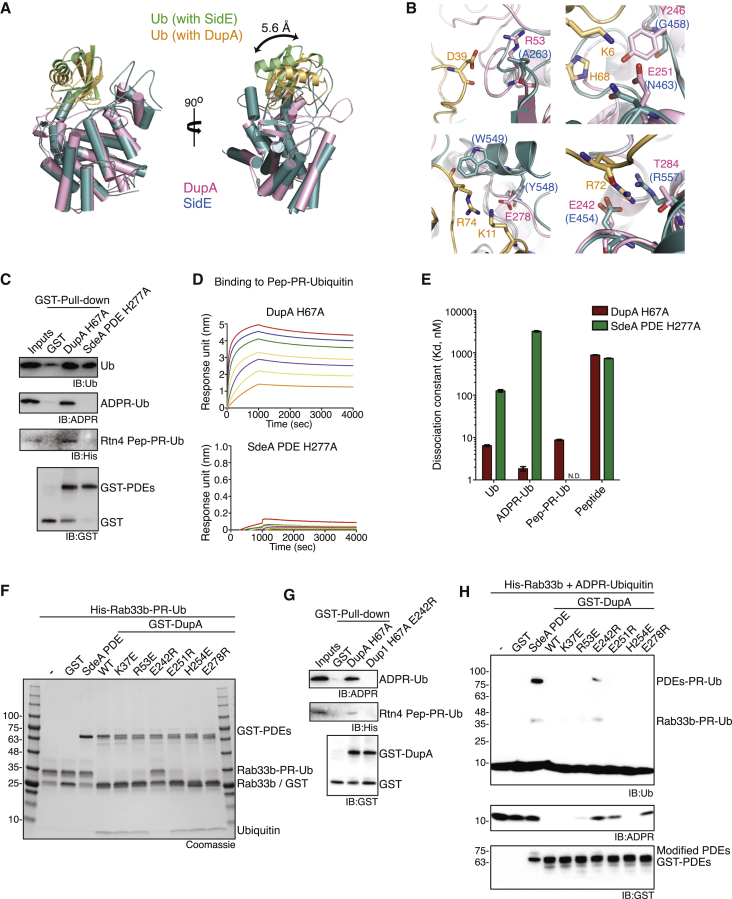
Crystal Structure and Binding Kinetics of DupA with Ubiquitin (A) Superimposition of the crystal structure of DupA-Ub complex with SidE PDE: Ub structure (PDB: 5ZQ3). (B) Electrostatic interactions between DupA (light pink) and Ub (light yellow). Interacting residues are represented as stick model and corresponding residues from SidE (light blue) are also shown. (C) GST pull-down between GST-DupA H67A mutant or GST-SdeA PDE H277A mutant and Ub, ADPR-Ub, or PR-ubiquitinated Rtn4 peptide. (D and E) Binding kinetics measurements of DupA H67A or SdeA PDE H277A. Sensograms of bio-layer interferometry (BLI) of catalytically inactive DupA_H67A_ (0.15–10 μM, orange to red) or SdeA PDE_H277A_ (1.5–100 μM, orange to red) to PR-ubiquitinated Rtn4 peptide. (D) Dissociation constants of DupA or SdeA PDE to various Ub species are presented as mean ± SEM. ^∗^SEM, standard error of mean. (E) Binding kinetics of SdeA PDE to PR-ubiquitinated Rtn4 peptide was not detectable (labeled N.D.). (F) PR-deubiquitination assays with DupA mutants. (G) GST pull-down assay between GST-DupA mutants and ADPR-Ub or PR-ubiquitinated Rtn4 peptide. (H) PR ubiquitination assays with DupA mutants. See also Figures S2, S3, S4, and Table S1.

To reveal the atomic basis underlying the bias of DupA toward deubiquitinase activity, we sought to determine the structure of enzymatically inactive DupA (H67A) in complex with a PR-ubiquitinated substrate. A Rtn4 peptide, acting as a minimal ubiquitination substrate of SdeA (Kalayil et al., 2018), was modified to harbor only one target serine residue (Figures S2A and S2B). Crystals of DupA H67A and PR-ubiquitinated Rtn4 peptide diffracted up to 2.0 Å, and molecular replacement revealed the densities for both Ub and DupA but not for the Rtn4 peptide, likely due to the flexibility of the peptide (PDB: 6RYA, Figure S2C). Superimposition of Ub in our DupA-Ub structure with the available structures of Ub complexed with the SidE PDE domain or DupB (PDB: 5ZQ3 and 6B7O, respectively) revealed a different orientation of Ub toward the conserved catalytic pocket (Figures 3A and S2D). In particular, DupA/B-Ub structures displayed a closed conformation with Ub due to extensive electrostatic interactions, while the SidE PDE domain lacked the corresponding residues (Figure 3B). Additionally, DupA H67A effectively interacted with Ub, ADPR-Ub, and PR-ubiquitinated substrates, whereas the SdeA PDE domain H277A only co-precipitated unmodified Ub (Figure 3C). Accordingly, DupA displayed a strong binding affinity and high k_on_ to Ub, ADPR-Ub, and PR-ubiquitinated peptides, while the SdeA PDE domain showed weak affinity to Ub and ADPR-Ub (Figures 3D, 3E, and S2E; Table 1). Unmodified Ub had similar residence time (1/k_off_) on both DupA and SdeA PDE domains; however, ADPR-Ub exhibited reduced residence time on the SdeA PDE domain (Figure S2F; Table 1). Moreover, the SdeA PDE domain did not bind to the PR-ubiquitinated serine peptide, whereas DupA maintained a strong binding affinity. These results were also confirmed by NMR titration (Figure S3).

**Table 1:** Binding Kinetics of DupA and SdeA PDE to Ub Species

		*k*_on_ ± SEM^a^ (10^2^ M^−1^s^−1^)	*k*_off_ ± SEM^a^ (10^−5^ s^−1^)	Residence Time (1/k_off_, min)	*K*_*d*_ ± SEM^a^ (nM)	R^2^^b^
DupA	Ub	102 ± 0.74	6.59 ± 0.29	253 ± 11	6.46 ± 0.29	0.99
	ADPR-Ub	188 ± 2.19	3.46 ± 0.41	482 ± 57	1.84 ± 0.22	0.97
	PR-ubiquitinated Rtn4 peptide	139 ± 1.31	12.2 ± 0.35	137 ± 3.9	8.75 ± 0.27	0.98
	Rtn4 Peptide	3.76 ± 0.04	33.4 ± 0.36	50 ± 0.5	887 ± 13.9	0.98
SdeA PDE	Ub	5.79 ± 0.06	7.34 ± 0.46	227 ± 14	127 ± 8.14	0.98
	ADPR-Ub	1.71 ± 0.05	55.0 ± 0.92	30 ± 0.5	3220 ± 104	0.96
	PR-ubiquitinated Rtn4 peptide	ND				
	Rtn4 Peptide	7.89 ± 0.10	58.4 ± 0.41	29 ± 0.2	740 ± 10.8	0.97

a SEM, standard error of mean

b R^2^, goodness of the curve fit between experimental data and mathematical 1:1 binding curve

To provide further insights into the dynamics of this reaction, we performed MD simulation of the PR-ubiquitinated Rtn4 peptide with either the DupA or SdeA PDE domains (Figure S4; Videos S1, S2, S3, and S4). We initiated the simulation by locating Ub outside the catalytic pocket via superimposing the PR-ubiquitinated peptide with the SidE-Ub complex structure (Figures S4A and S4B). After 100 ns of simulation with DupA, Ub translocated and settled down in the catalytic pocket, whereas Ub did not find the catalytic pocket during the entire simulation (5 μs) with the SdeA PDE domain. Placing the PR-ubiquitinated peptide closer to the catalytic pocket of SdeA PDE domain resulted in a short residence time for both Ub and the peptide in the catalytic pocket, whereas the catalytic histidine of DupA remained close to the phosphate on the PR-ubiquitinated substrate throughout the simulation (5 μs) (Figures S4C and S4D). Based on these observations, we postulated that the differences in binding dynamics and affinities of DupA and SdeA to Ub might help explain how two similar PDE domains elicit two counteracting reactions. To explore this, we introduced multiple mutations in the DupA PDE domain (Figure 3F). Only the DupA E242R mutant affected the hydrolase ability and displayed reduced binding affinity to PR-ubiquitinated substrates (Figure 3G). More importantly, the same mutant was now able to promote stable PR ubiquitination (Figure 3H). These data reveal that the extent of interaction between PDE domains and Ub governs the directionality of their enzymatic activity. The hydrolase (PR deubiquitinase) activity is favored by high affinity and longer residence time of the PR-ubiquitinated substrate to DupA/B, while the transferase (PR-Ub ligase) activity is dictated by lower-affinity interactions of the SidE family PDE domain with ADPR-Ub.

### Identification of PR-Ubiquitinated Substrates upon *Legionella* Infection

Until now, no general workflow for the specific enrichment of PR-ubiquitinated proteins has been presented. Initial concepts relied on the use of tagged Ub that lacks the C-terminal GG motif (Ub^ΔGG^) and can only be attached to other proteins by PR ubiquitination. However, this concept has several drawbacks and limitations, as it relies on overexpression of tagged Ub, which might stress the cell and is only usable in genetically engineered or transfected cells. Therefore, we aimed to establish a protocol that enables the efficient enrichment of PR-ubiquitinated proteins that do not rely on genetic perturbations.

**Figure 4:**
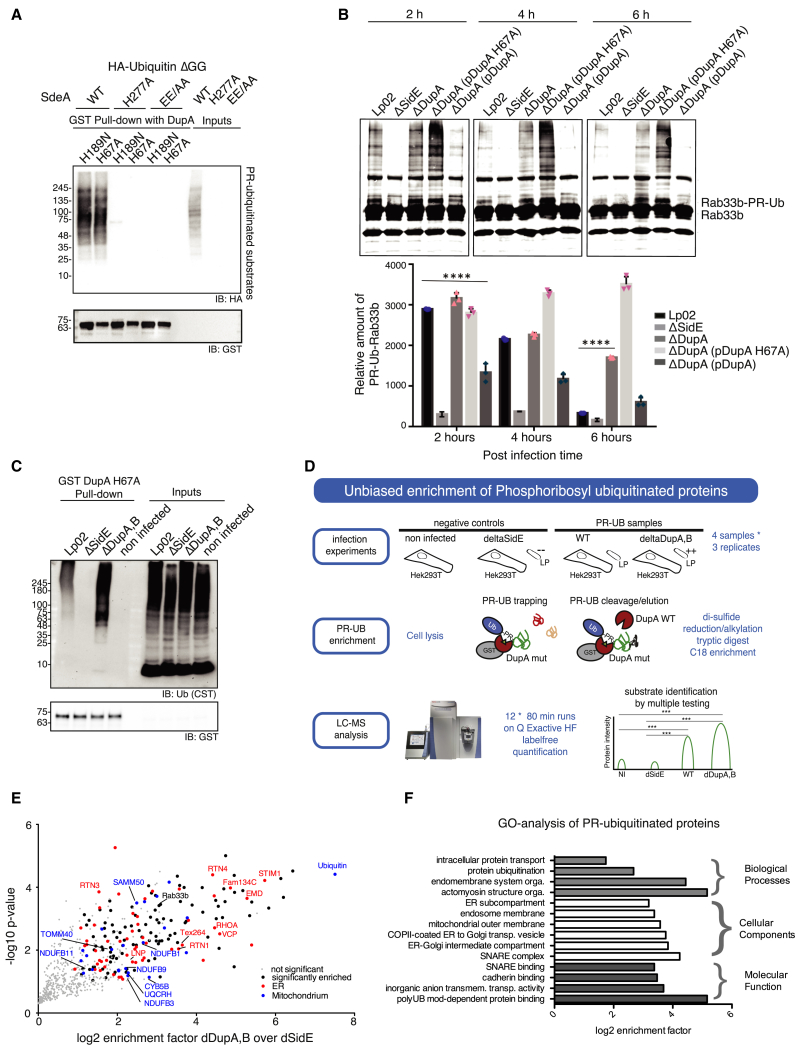
Proteomic Platform to Identify Phosphoribosyl-Ubiquitinated Substrates (A) Trapping PR-ubiquitinated substrates with catalytically inactive DupA mutants. Lysates of cells expressing SdeA and Ub without two C-terminal glycine residues were incubated with DupA mutants to enrich the PR-ubiquitinated substrates. (B) Enrichment of PR-ubiquitinated substrates from cells infected with *Legionella* strains. PR ubiquitination of Rab33b was monitored through the infection as indicated. Data are represented as mean ± SD. (C) Trapping endogenous PR-ubiquitinated substrates from cells infected with *Legionella*. Lysates of cells infected with indicated strains were incubated with DupA trapping mutant (H67A) and analyzed for enrichment of ubiquitinated proteins. (D) Schematic diagram of novel proteomic approach for identifying PR-ubiquitinated substrates. (E) A single-sided volcano plot depicting identified PR-ubiquitinated substrates by quantitative mass spectrometry. (F) Gene Ontology (GO) analysis of PR-ubiquitinated proteins. See also Figure S5 and Table S2.

Given that DupA displayed strong binding affinity to PR-ubiquitinated substrates, we hypothesized that catalytic inactive mutants of DupA (H67A or H189N) could be used as trapping mutants to enrich PR-ubiquitinated proteins from cellular lysates for subsequent proteomic analysis. To test this, we first co-expressed SdeA together with HA-tagged Ub 1-74 (HA-Ub^ΔGG^), which lacks two glycine residues at the carboxy terminus, thereby preventing canonical ubiquitination. SdeA utilizes HA-Ub^ΔGG^ to catalyze ubiquitination of cellular proteins, as demonstrated by a smear of PR-ubiquitinated proteins in cells (Figure 4A). Importantly, both DupA inactive mutants (H67A or DupA H189N) effectively bound and enriched PR-ubiquitinated substrates from cells co-expressing Ub^ΔGG^ and WT SdeA, but not canonical ubiquitinated proteins from cells expressing mutant SdeA (H277A or EE/AA, Figures 4A and S5A). Interestingly, DupA H67A mutant enriched ADPR-Ub and ADPR-Ub-conjugated proteins from cells expressing SdeA (H277A), while H189N mutant could not. Moreover, the enriched PR-ubiquitinated substrates on DupA-trapping mutants could be cleaved *in vitro* by incubation with WT DupA (Figure S5B).

Next, we sought to identify endogenous substrates in *Legionella*-infected cells. We first established and tested different *Legionella* strains for their ability to modulate PR-ubiquitinated substrates in infected cells. Infection with WT *Legionella* strain (Lp02) showed maximum PR ubiquitination of Rab33b at 2 h post-infection and subsequent reduction, while a strain lacking DupA (*ΔdupA*) maintained the PR ubiquitination up to 6 h post-infection (Figure 4B). Reconstitution of the *ΔdupA* strain with the DupA H67A mutant led to a slight increase in endogenous Rab33b PR ubiquitination , suggesting that DupA H67A acts as a dominant-negative mutant in infected cells. Moreover, deletion of DupB (*ΔdupB)* or both DupA and DupB (*ΔdupA/B*) led to an increased and more prolonged endogenous Rab33b PR ubiquitination 4–6 h post-infection (Figure S5C). This indicates that DupA and DupB may regulate PR ubiquitination at different stages of infection. Deletion of both DupA and DupB had no significant effect on overall *Legionella* proliferation in cultured cells (Figure S5D).

In order to identify endogenously modified proteins, we used label-free MS quantification and examined the differential enrichment of PR-ubiquitinated proteins under the different infection conditions. We enriched PR-ubiquitinated proteins from cells infected with WT *Legionella* or the *ΔdupA/B* strains by GST-DupA H67A pull-down (Figure 4C). Eluted PR-ubiquitinated proteins released from the beads by cleaving the phosphodiester bond to the substrate serine using DupA (WT). Due to this non-denaturing elution strategy, only PR-ubiquitinated proteins and not unspecific binders, such as canonically ubiquitinated proteins, were eluted and further analyzed. We reproducibly quantified more than 1,000 proteins from cells infected with WT *Legionella* or the *ΔdupA/B* strain by using the DupA H67A-trapping mutant (Figures 4C and 4D; Table S2). Of these, 181 proteins were consistently and significantly enriched in cells infected with PR-ubiquitinating bacteria (WT or *ΔdupA/B*) compared to non-infected or cells infected with a SidE-deficient (*ΔsidE*) strain (Figure 4E; Table S2). The mutant strain proliferated as similar to WT *Legionella*. A gene ontology (GO)-term analysis of newly identified PR-ubiquitinated proteins suggest that these proteins are present in common interaction networks, linked functionally, and can regulate several cellular pathways (Figure 4F). Among the identified PR-ubiquitinated proteins, a number of endoplasmic reticulum (ER) resident proteins (FAM134C, RTN1, RTN3, RTN4, lunapark 1 [LNP1], and TEX264) were scored at high ratios (Table S2; Figure 4E). Most of these proteins belong to a group of reticulon-type ER membrane proteins that are implicated in regulation of ER remodeling or selective ER fragmentation and autophagy (ER-phagy) (Grumati et al., 2017, Khaminets et al., 2015). In addition, proteomic analysis also identified proteins related to other cellular pathways, including mitochondrial proteins, Golgi components, autophagy, edocytic trafficking, and the proteasome (Table S2). In addition, proteomic analysis also identified proteins related to other cellular pathways like mitochondrial metabolism, autophagy, and the proteasome.

### Multiple ER Proteins Are PR-Ubiquitinated during *Legionella* Infection

**Figure 5:**
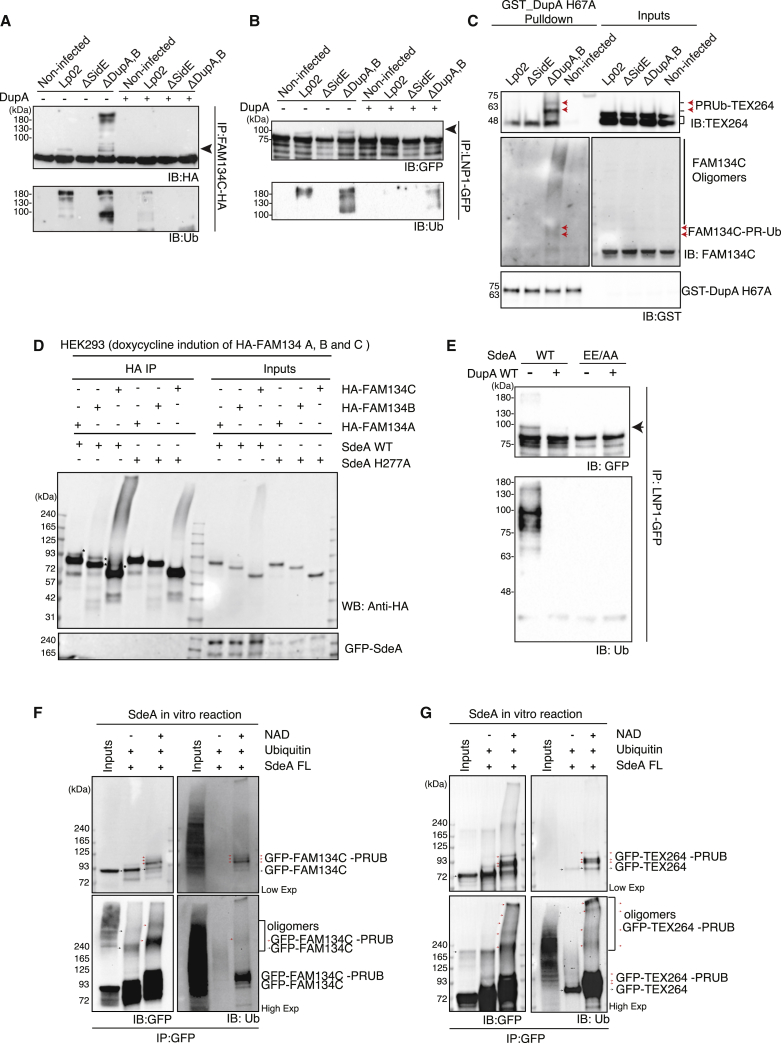
Validation of PR Ubiquitination on Multiple ER Proteins (A) HEK293T cells expressing HA-tagged FAM134C were infected with *Legionella* strains for 2 h, followed by enrichment of FAM134C from lysates using HA beads. Immunoprecipitated products were treated with or without Dup1 for 30 min at 37°C followed by immunoblotting. (B) HEK293T cells expressing GFP-tagged LNP1 were infected with *Legionella* strains for 2 h, immunoprecipitated with anti-GFP agarose, treated with Dup1 similar to (A), and subjected to immunoblotting. (C) Enrichment of PR-ubiquitinated endogenous FAM134C and TEX264 with GST-tagged H67A Dup1 upon infection. (D) HEK293T cells expressing HA-tagged FAM134A, FAM134B, and FAM134C under a doxycycline promoter were transfected with SdeA or its PDE mutant SdeA (H277A). Cells were lysed and lysates were used to immunoprecipitate FAM134 using HA beads followed by western blotting. (E) PR ubiquitination of LNP1-GFP in HEK293T cells co-transfected with LNP1-GFP and SdeA or SdeA (EE/AA). (F and G) *In vitro* PR ubiquitination reaction of GFP-FAM134C and GFP-TEX264, respectively. See also Figure S6.

We next attempted to validate PR ubiquitination of selected ER proteins upon *Legionella* infection. First, we observed that HA-FAM134C and LNP1-GFP were PR-ubiquitinated in cells infected with WT but not in non-infected cells or cells infected with a SidE-deficient (*ΔsidE*) strain (Figures 5A and 5B). This PR ubiquitination was significantly more pronounced in cells infected with *ΔdupA/B* (Figures 5A and 5B). This is similar to the effect observed for PR ubiquitination of Rab33b, a known SidE substrate (Figure 4B). Furthermore, these mobility shifts were completely removed when the lysates of infected cells were pre-incubated with purified DupA (Figures 5A and 5B). In addition, endogenous FAM134C and TEX264 isolated from cells infected with *ΔdupA/B* strain were significantly enriched in pull-down assays with DupA H67A-trapping matrix (Figure 5C). Heterologous expression of WT SdeA, but not the inactive PDE mutant (SdeA H277A), in HEK293T cells promoted PR ubiquitination of all three FAM134 family isoforms (FAM134A, FAM134B, and FAM134C) as well as LNP1-GFP (Figures 5D and 5E). Along with these biochemical analyses, we observed high-molecular-weight species of FAM134C (Figures 5A, 5C, and 5D), which are also dependent on the cleavage with DupA (Figures 5A). This suggested that PR ubiquitination of FAM134 or TEX264 proteins may participate in formation or stabilization of FAM134C oligomers. Indeed, in *ΔdupA,B*-infected cells where the PR ubiquitination is more enhanced, the extent of oligomer formation is more prominent than in WT *Legionella*-infected cells where PR ubiquitination is more transient (Figure 5A). Similarly, high-molecular-weight oligomers of FAM134C and FAM134B are also observed in SdeA-transfected cells (Figures S6A and S6B). This increase in oligomerization triggered by SdeA-mediated PR ubiquitination was further validated by co-immunoprecipitation of FAM134C-GFP and FAM134C-HA proteins (Figure S6C). In order to confirm that these effects are direct consequences of PR ubiquitination , we performed an *in vitro* PR ubiquitination assay on GFP-FAM134C and GFP-TEX264 in presence of purified SdeA. Both proteins were strongly modified by SdeA *in vitro* as detected by shifted bands corresponding to different PR-ubiquitinated forms of the proteins (Figures 5F and 5G). By using mass spectrometric analysis of *in vitro*-modified TEX264, we were able to identify serine 239 of TEX264 as a direct site of PR ubiquitination by SdeA. Taken together, these results validate PR ubiquitination of multiple ER proteins that are identified by DupA-trapping mutant proteomic matrix.

### PR Ubiquitination Causes ER Remodeling and Vesicle Recruitment to Bacteria

The ER has a central role in *Legionella* infection as it is the main source of membranes that form LCVs (Steiner et al., 2018). Previous work indicated that RTN4 may be a critical substrate for this pathway (Kotewicz et al., 2017), yet the current knowledge of signals that mediate such dynamic changes in ER fragments are poorly understood. Since we identified and validated PR ubiquitination on multiple ER proteins, we wondered whether PR ubiquitination can affect ER remodeling and dynamics.

**Figure 6:**
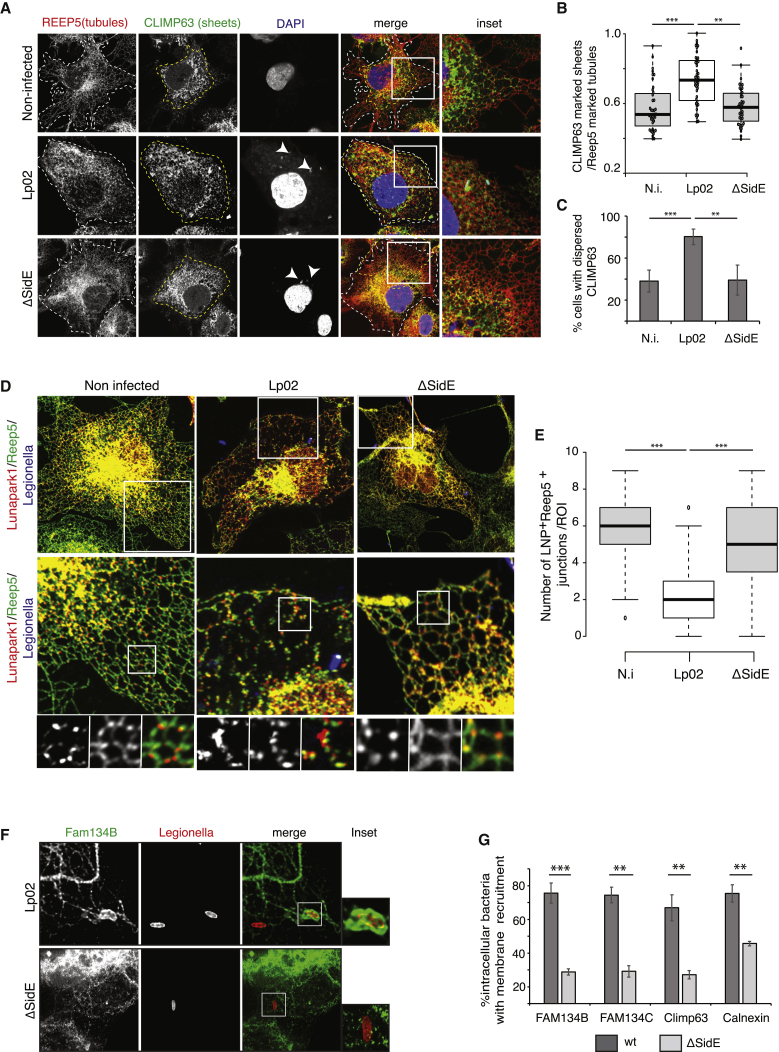
Roles of PR Ubiquitination in ER Remodeling and Fragmentation (A) A549 cells were infected with WT and *ΔsidE Legionella* strains. 2 h post-infection cells were fixed and coimmunostained using Reep5 and CLIMP63 antibodies to mark ER tubules and ER sheets, respectively. DAPI marks nucleus and cytosolic bacteria. (B) Images in (A) were used to quantitate area occupied by ER sheets and ER tubules. Ratios of these values are plotted for non-infected, WT, and *ΔsidE* strains. Data represent 40 cells taken from three independent experiments. (C) Cells in (A) with area ratio >0.6 are classified as cells with dispersed ER sheets. Data represent 40 cells taken from three independent experiments. (D) *Legionella*-infected A549 cells were fixed and coimmunostained with antibodies against Reep5 and LNP1 to mark ER tubules as three-way junctions. DAPI marks nucleus and cytosolic bacteria. (E) Images in (D) were used to quantitate number of LNP1/Reep5 positive junctions per 50 μm^2^ regions of interest (ROIs) chosen from the cell periphery. (F) *Legionella*-infected A549 cells expressing FAM134B-HA were coimmunostained with antibodies against *Legionella* and HA followed by simulated emission depletion (STED)imaging. (G) Recruitment of membrane markers to *Legionella* was quantitated 2 h after infection in A549 cells. Error bars indicate standard deviation, ^∗∗∗^p < 0.001 by two-tailed type III Student’s t test. See also Figure S7.

Initially, we showed that overexpression of SdeA WT, but not its mART mutant (SdeA EE/AA), leads to fragmentation of FAM134B-labeled ER networks (Figure S7A). Similarly, in cells infected with WT *Legionella*, the FAM134B-GFP-labeled ER was more fragmented than in cells infected with *ΔsidE Legionella*. Furthermore, in non-infected cells, the ER forms a dense meshwork with highly branched structures, while cells infected with *Legionella* showed a fragmented ER with larger spaces between ER tubules. Interestingly, cells infected with *ΔsidE Legionella* strain showed an intermediate phenotype between WT and non-infected cells (Videos S5 and S6; Figure S7B). Next, to check the effect of SidE on ER sheets and tubules, we coimmunostained the ER tubule (Reep5) and the ER sheet (CLIMP63) markers in infected A549 cells where Reep5 staining showed a highly branched tubular network and CLIMP63 marks the central sheet-like ER. In contrast, upon infection with WT *Legionella*, both the tubular ER and the ER sheets appeared fragmented compared to non-infected cells or *ΔsidE*-strain-infected cells (Figure 6A). Also, the CLIMP63 staining was more dispersed, and separation between the ER tubules and sheets were less clear (Figures 6B and 6C).

Video S5. ER Dynamics in Live Cells Expressing SdeA-mcherry and FAM134B-GFP, Related to Figure 6 Dynamics of ER structure under PR-ubiquitinating condition (SdeA-WT-mcherry) is monitored by imaging FAM134B-GFP.

Video S6. ER Dynamics in Live Cells Expressing SdeA (EE/AA)-mcherry and FAM134B-GFP, Related to Figure 6 Dynamics of ER structure under non-PR-ubiquitinating condition (SdeA-EE/AA-mcherry) is monitored by imaging FAM134B-GFP.

We next analyzed the specialized three-way ER junctions that are regulated by actions of LNP1 and atlastins. Overexpression of LNP1 stabilizes ER junctions, while its depletion causes expansion of ER sheets (Wang et al., 2016). LNP1 and Reep5 in *Legionella*-infected cells were localized on fragmented ER tubules in WT *Legionella*-infected cells, while LNP1 localized to three-way junctions in non-infected cells or *ΔsidE*-strain-infected cells (Figures 6D and 6E). Subsequently, we performed an interactome analysis of GFP-LNP1 in cells expressing SdeA versus those with SdeA (EE/AA). In SdeA-expressing cells, the LNP1 interactome showed reduced interactions with other ER proteins, like Derlin1, YIF1, GOT1B, and also the endosomal protein DNAJC13 compared to SdeA (EE/AA)-expressing cells (Figure S7C). This suggests that PR ubiquitination of LNP1 may affect its interactions with other ER proteins and/or its localization in maintaining three-way junctions, which are both critical steps in ER tubule remodeling.

Other ER membrane proteins, like FAM134B, FAM134C, and CLIMP63, are also recruited to bacteria upon infection with WT bacteria (Figures 6F, 6G, and S7D). CLIMP63-positive ER membranes formed a coat around the bacteria in WT *Legionella*-infected cells, which was significantly reduced in *ΔsidE*-infected cells (Figure 5D). Similarly, WT *Legionella*-infected cells showed strong recruitment of FAM134C-positive ER fragments to LCV (Figure S7D). In addition, WT *Legionella* appeared to be completely surrounded in a FAM134B shell by 2 h post-infection (Figure 6F). The recruitment of ER membrane proteins (FAM134B, FAM134C, CLIMP63, calnexin) to bacteria was severely reduced in *ΔsidE*-infected cells (Figure 6G). These observations collectively indicate that SidE-mediated serine ubiquitination is involved in regulating ER remodeling and recruiting ER membranes to bacteria vacuoles.

## Discussion

This study identifies an uncharacterized role of the PDE-domain-containing *Legionella* effector proteins DupA/LaiE and DupB/LaiF as PR-Ub specific deubiquitinases. They are essential and complementary enzymes to control the balance of PR ubiquitination of multiple substrates upon bacterial infection. DupA and DupB specifically catalyze deubiquitination of PR-Ub via their PDE domains, which are structurally indistinguishable from the PDE domains of SidE enzymes that are known to mediate PR ubiquitination. These contrasting effects depend on the substrates served to DupA/B and their kinetic parameters. While PDE domains of SidE enzymes do not bind to PR-ubiquitinated substrates and have moderate binding affinity to Ub, DupA and DupB show strong k_on_ rates to Ub and selective affinity to PR-ubiquitinated peptides. In fact, by weakening the affinity of the PDE domain to Ub-ubiquitinated peptides, the DupA/B PDE domains can be converted into SidE-type Ub ligases. A similar phenomenon is observed with the regulation of prokaryotic ubiquitin-like protein (Pup) by PafA (Pup ligase) and Dop (depupylase) family members, which have highly homologous catalytic domains, yet they mediate chemically opposite reactions (Imkamp et al., 2010, Pearce et al., 2008). Moreover, PafA and Dop share a conserved Pup binding site, similar to what we demonstrate with DupA/B and PDE domain of SidE enzymes. In addition, Dop has strong binding affinity to Pup and mediates depupylation of substrates, while PafA displays weaker binding to Pup and catalyzes formation of an isopeptide bond between Pup and lysine of substrates (Özcelik et al., 2012). Together, this supports the notion that formation of stable enzyme:substrate complexes might be required to mediate the cleavage reaction. In contrast, the ligation reaction requires moderate binding affinity to substrates for releasing the newly synthesized final products.

Our findings also provide critical insights into the functional roles of *Legionella*-mediated PR ubiquitination during bacterial infection. The DupA-mutant-mediated trapping strategy revealed more than 180 proteins that are potentially PR-ubiquitinated by *Legionella.* Analysis of the PR-ubiquitinome from *Legionella*-infected cells showed that substrates of SidE have various cellular functions, including the regulation of ER remodeling and recruitment of ER membranes to the LCVs in which bacteria reside and proliferate (Qiu and Luo, 2017, Robinson and Roy, 2006, Steiner et al., 2018, Swanson and Isberg, 1995). Deletion of all four SidE family enzymes (*ΔsidE*) has also been shown to impair ER recruitment to LCVs (Bardill et al., 2005, Luo and Isberg, 2004). Identification of ER reticulon domain-containing proteins as prominent SidE family substrates is thus of particular interest. Previously, RTN4 was considered to be the chief target of PR ubiquitination , which led to rearrangement of ER tubules, yet the mechanism of action of RTN4 remains unknown (Kotewicz et al., 2017). Here, we show that multiple ER regulatory proteins are PR-ubiquitinated upon infection and may act together during this process. Among them are the FAM134 family proteins (A, B, and C) and the TEX264 and RTN3 proteins, which are capable of fragmenting ER and subsequently delivering ER pieces to the lysosome for degradation via the ER-phagy pathway (An et al., 2019, Chino et al., 2019, Dikic, 2018, Grumati et al., 2017, Khaminets et al., 2015). Previous studies have shown that overexpression and subsequent oligomerization of reticulon proteins may play a critical role in triggering the ability of FAM134 and RTN3 to fragment ER (Grumati et al., 2017, Khaminets et al., 2015). This may be assisted with the special arrangement of transmembrane insertions of the reticulon domain that are predisposing lipid bilayers for membrane curvature (Bhaskara et al., 2019). Interestingly, oligomerization of FAM134C and TEX264 correlate with their PR ubiquitination. We speculate that PR ubiquitination may induce conformational changes that favor their oligomerization or that multiple ubiquitination moieties may attract other proteins and stabilize such multimeric complexes.

Also, other proteins responsible for maintaining the ER framework, like lunapark and atlastins, which are implicated in ER tubule branching, are PR-ubiquitinated by SidE proteins (Steiner et al., 2017, Wang et al., 2016). Time-lapse imaging of SdeA-transfected cells shows slower dynamics of FAM134B-labeled ER tubules compared to SdeA(EE/AA) (Videos S5 and S6). In WT *Legionella*-infected cells, there are fewer interconnections between adjacent tubules compared to those seen in non-infected or *ΔsidE*-infected cells. These effects may be due to alterations in the functions of proteins like lunapark or atlastins, which help to bridge ER tubules to form a meshwork. It will be interesting to study in the future why these fragments are specifically recruited to the LCV and not targeted by the host autophagic pathway. This might be a result of the combinatorial effect of other bacterial effectors such as the cysteine protease, RavZ (Choy et al., 2012), the serine protease Lpg1137 (Arasaki et al., 2017), and the spingosine-1 phosphate lyase *Lp*Sp1 (Rolando et al., 2016) that were all shown to inhibit autophagy at different stages of the autophagic pathway.

Taken together, DUPs appear to predominantly regulate the dynamics of PR ubiquitination catalyzed by SidE enzymes *in vivo*. In cells infected with the WT *Legionella* strain, maximum PR ubiquitination of the SidE substrates occurs 1–2 h post-infection and largely decreases after 6 h. On the other hand, deletion of DupA and DupB in *Legionella* increased and prolonged the level of ubiquitination of substrates such as FAM134C and Rab33. Further studies to see the extent of ER fragmentation from cells infected with *Legionella* strain without both DUPs will give us more understanding about the PR ubiquitination -dependent ER fragmentation. Intriguingly, another *Legionella* effector, SidJ has also been shown to negatively regulate SidE-mediated PR ubiquitination. Instead of targeting substrates, SidJ was shown to be an inhibitor of SidE ligases by mediating glutamylation of the mART domain, which blocks the production of ADPR-Ub and the entire PR ubiquitination process (Bhogaraju et al., 2019, Black et al., 2019, Gan et al., 2019). It appears, therefore, that *Legionella* tightly regulates the level of PR ubiquitination in cells, as this is toxic in both yeast and mammalian cells (Bhogaraju et al., 2016). The PR-ubiquitinome analyses also identified proteins that could potentially regulate autophagy, Golgi, or mitochondrial dynamics, indicating that PR ubiquitination may have broad and pleotropic effects on host cell responses. Understanding the mechanistic details of PR ubiquitination of individual proteins may pave the way for more exciting discoveries in the field.

## STAR★Methods

### Key Resources Table

**Table:** 

REAGENT or RESOURCE	SOURCE	IDENTIFIER
**Antibodies**		

Ubiquitin Ubi-1	abcam	Cat# ab7254; RRID:AB_305802
Ubiquitin	Cell Signaling Technology	Cat# 3936S; RRID: AB_331292
HA	Cell Signaling Technology	Cat# 2367S; RRID: AB_10691311
GST	Cell Signaling Technology	Cat# 2625S; RRID:AB_490796
His_6_	Roche	Cat# 11922416001; RRID: AB_514486
ADPR	Millipore	Cat# MABE1016; RRID: AB_2665466
HA	Santa cruz biotechnology	Cat# Sc-7392; RRID: AB_627809
Myc	Santa Cruz Biotechnology	Cat# Sc-42; RRID: AB_2282408
GAPDH	Cell Signaling Technology	Cat# 2118S; RRID: AB_561053
Rtn4	Cell Signaling Technology	Cat# 13401S; RRID: AB_2798209
*Legionella*	abcam	Cat# Ab20943; RRID: AB_445931

**Chemicals, Peptides, and Recombinant Proteins**		

NAD	Sigma-Aldrich	N0632
Rtn4 Peptide 1	Genscript	N/A
Rtn4 Peptide 2	Genscript	N/A
Rtn4 Peptide 3	Genscript	N/A
Rtn4 Peptide 4	Genscript	N/A
Rtn4 Peptide 5	Genscript	N/A
USP2	Dikic lab	N/A

**Critical Commercial Assays**		

Pro-Q diamond staining	Thermo Fisher Scientific	P33300

**Deposited Data**		

Atomic coordinates (Dup1_4-345_)	This study	PDB: 6RYB
Atomic coordinates (Dup1_4-345_:Ub complex)	This study	PDB: 6RYA
Unprocessed data from this manuscript at Mendeley Data	This study	https://doi.org/10.17632/bkwjctz23n.1

**Experimental Models: Cell Lines**		

HeLa	ATCC	Cat# CCL-2; RRID: CVCL_0030
HEK293T	ATCC	Cat# CRL-3216; RRID: CVCL_0063
A549	ATCC	N/A

**Experimental Models: Organisms/Strains**		

*Eschericha coli* T7 express	New England Biolabs	C2566H
*Eschericha coli* Turbo	New England Biolabs	C2984H
*Legionella pneumophila* lp02 WT	Zhao-Qing Luo	N/A
*Legionella pneumophila* ΔSidE	Zhao-Qing Luo	N/A
*Legionella pneumophila* ΔDupA	Zhao-Qing Luo	N/A
*Legionella pneumophila* ΔDupB	Zhao-Qing Luo	N/A
*Legionella pneumophila* ΔDupA/B	Zhao-Qing Luo	N/A

**Recombinant DNA**		

pParallel GST2-DupA WT	This study	N/A
pParallel GST2-DupA H67A	This study	N/A
pParallel GST2-DupA H189N	This study	N/A
pParallel GST2-DupA E126A	This study	N/A
pParallel GST2-DupA E242R	This study	N/A
pParallel GST2-DupA 4-345 WT	This study	N/A
pParallel GST2-DupA 4-345 H67A	This study	N/A
pParallel GST2-DupB WT	This study	N/A
pParallel GST2-DupB H67A	This study	N/A
pParallel GST2-DupB H189N	This study	N/A
pParallel GST2-DupB E126A	This study	N/A
pParallel GST2-SdeA (222-593) WT	This study	N/A
pParallel GST2-SdeA (222-593) H277A	This study	N/A
pParallel GST2-SdeB (222-592)	This study	N/A
pParallel GST2-SdeC (222-592)	This study	N/A
pParallel GST2-SidE (219-591)	This study	N/A
pParallel GST2-Lpg1496 (297-Cterm)	This study	N/A
pParallel GST2-Lpg2523 (492-Cterm)	This study	N/A
pET21a-Rab33b	Dikic lab	N/A
pHAC1-Ub ΔGG	This study	N/A
pHAC1-SdeA	This study	N/A
pHAC1-SdeA EE/AA	This study	N/A
pGFPC1-SdeA WT	This study	N/A
pGFPC1-SdeA EE/AA	This study	N/A

**Software and Algorithms**		

HHpred	(Alva et al., 2016)	https://toolkit.tuebingen.mpg.de/hhpred
XDS	(Kabsch, 2010)	http://xds.mpimf-heidelberg.mpg.de/
CCP4	(Winn et al., 2011)	https://www.ccp4.ac.uk/
Phenix	(Adams et al., 2010)	https://www.phenix-online.org/
Coot	(Emsley and Cowtan, 2004)	https://www2.mrc-lmb.cam.ac.uk/personal/ pemsley/coot/
Pymol	The PyMOL Molecular Graphics System, Version 1.7.6.0, Schrodinger, LLC	https://pymol.org/2/
GraphPad Prism 5.0	GraphPad Software	https://www.graphpad.com/
FIJI	(Schindelin et al., 2012)	https://fiji.sc/

### Lead Contact and Materials Availability

Further information and requests for reagents may be directed to and will be fulfilled by the Lead Contact Ivan Dikic (ivan.dikic@biochem2.de). All unique/stable reagents generated in this study are available from the Lead Contact with a completed materials transfer agreement (MTA).

### Experimental Model and Subject Details

#### Microbe strains

*E. Coli* strains chemically competent were used in this study.

1. T7 express (NEB, C2566H).

2. NEB Turbo (NEB, C2984H).

These cells were stored at **-**80°C and grown in LB medium at 37°C. T7 express cells were used for recombinant protein expression. NEB Turbo cells were used for plasmid amplification and molecular cloning.

*Legionella pneumophila* strains used in this study.

1. Lp02 (WT strain of *Legionella*)

2. ΔSidE strain lacking all 4 members of SidE family

3. ΔSidE strain complemented with WT SdeA

4. ΔSidE strain complemented with SdeA EE/AA mutant

*L. pneumophila* strains were grown for 3 days on N-(2-acetamido)-2-amino-ethanesulfonic acid (ACES)-buffered charcoal-yeast (BCYE) extract agar, at 37°C.

#### Cell lines

HeLa, HEK293T and A549 cells were cultured in DMEM supplemented with 10% FBS, 100 I.U./mL penicillin and 100 mg/mL streptomycin (Pen/Strep) at 37°C, 5% CO_2_. Raw264.7 macrophages were cultured in RPMI supplemented with 10% FBS. Detailed information of cell Lines, strains are provided in Key Resources Table.

CO_2_. Raw264.7 macrophages were cultured in RPMI supplemented with 10% FBS.

### Method Details

#### *Legionella pneumophila* culture and infection

*L. pneumophila* strains were grown for 3 days on N-(2-acetamido)-2-amino-ethanesulfonic acid (ACES)-buffered charcoal-yeast (BCYE) extract agar, at 37°C, followed by growth for 20 h in CYE media. Bacterial cultures of optical density between 3.2-3.6 were used to infect cells at an MOI of 1:10. HEK293T cells used for infection were transfected with CD32 to facilitate infection of cells with *Legionella*.

#### Protein expression and purification

Expression and purification of Rab33b have been previously described (Bhogaraju et al., 2016, Qiu et al., 2016). For PDE domains, SdeA_222-593_, SdeB_222-592_, SdeC_222-592_, SidE_219-591_, DupA, DupB, lpg1496_297-Cterm_, lpg2523_492-Cterm_ and DupA_4-345_ (crystallized construct) were cloned into pParallelGST2 vector (Sheffield et al., 1999). Full length SdeA is cloned into pGEX-6P-1 vector. T7 express *E.coli* competent cells (NEB) were transformed with plasmids and grown in LB medium to an OD_600_ of 0.6-0.8 at 37°C. Protein expression was induced by addition of 0.5 mM IPTG (isopropyl _D_-thiogalactopyranoside) and cells were further grown overnight at 18°C and harvested. The cell pellet was resuspended in lysis buffer (50 mM Tris-HCl pH 7.5 and 150 mM NaCl, 1 mM TCEP) and lysed by sonication and centrifuged at 13,000 rpm to clarify the supernatant. The supernatant was incubated 1 h with glutathione-*S*-Sepharose pre-equilibrated with washing buffer (50 mM Tris-HCl pH 7.5, 500 mM NaCl) and non-specific proteins were cleared with washing. GST-proteins were eluted with elution buffer (50 mM Tris-HCl pH 8.0, 50 mM NaCl, 15 mM reduced glutathione) and buffer exchanged to storage buffer (50 mM Tris-HCl pH 7.5, 150 mM NaCl). For DupA_4-345_ WT, H67A or H67A/H189N, instead of using elution buffer, glutathione beads were incubated with sfGFP-TEV protease (Wu et al., 2009) overnight at 4°C. Eluted proteins were further purified by anion exchange chromatography on HitrapQ (GE Healthcare) and fractions contacting samples were loaded onto size exclusion column (Superdex 75 16/60, GE Healthcare) pre-equilibrated with 50 mM Tris-HCl pH 7.5, 50 mM NaCl, 1 mM TCEP. Proteins were concentrated to 10 mg/mL and stored for crystallization. PR-ubiquitinated Rtn4 peptide were synthesized by incubation with GST-SdeA_FL_, NAD, Ub in the reaction buffer (50 mM Tris-HCl pH 7.5, 150 mM NaCl) at 37°C for 2 h. To effectively modify the peptide, Ub were iteratively added to reaction mixture every 30 min (Shin et al., 2017). PR-ubiquitinated peptide were further purified with Ni-NTA agarose and size exclusion chromatography (Superdex 30 prep grade 16/60, GE Healthcare).

#### Crystallization

The concentrated DupA_4-345_ WT were screened with sitting drop matrix screens in 96-well plate with 150 nL of protein and 150 nL of precipitant solution at 293K. Diffraction quality crystal appeared from solution containing 18 - 20% PEG3350 / PEG4000, 100 mM HEPES pH 7.5, 200 mM Ammonium sulfate. For complex crystal of DupA_4-345_ H67A:PR-ubiquitinated Rtn4, DupA is mixed with PR-ub-peptide at 1:5 molar ratio and concentrated to 10 mg/mL. Crystals were obtained from solution containing 20 - 22.5% PEG 3350/ PEG4000, 100 mM Tris-HCl pH 8.0, 100 mM Magnesium chloride.

#### Data **collection, processing and structure** determination

Crystals were cryo-protected using mother liquor solution supplemented with 15% (v/v) glycerol. Diffraction data were collected on single frozen crystal in a nitrogen stream at 100K at beamline PXI as Swiss Light Source, Villigen. Initial datasets were processed using XDS (Kabsch, 2010), and phases were determined by Phaser molecular replacement in ccp4 module with SdeD, SdeD:Ub as template model (McCoy et al., 2007), PDB:6B7P, 6B7O, respectively). Structure refinement and manual model building were performed with Coot and Phenix.Refine (Afonine et al., 2012, Emsley et al., 2010). During data analysis, twinning is indicated from DupA:PR-Rtn4-peptide dataset, and twinning operators were applied during refinement.

#### *In vitro* ubiquitination/deubiquitination assays

SdeA mediated PR ubiquitination assay was done as previously described (Kalayil et al., 2018). Briefly, 2 μg of purified Rab33b, ADPR-ubiquitin, 200 μM of NAD^+^ were incubated with 1 μg of GST-PDEs at 37°C for 1 h in 30 μl of reaction buffer (50 mM Tris-HCl pH 7.5, 150 mM NaCl). Deubiquitination assay were performed by mixing 1 μg PR-ubiquitinated Rab33b with 1 μg of GST-PDEs at 37°C for 1 h in 30 μl of reaction buffer (50 mM Tris-HCl pH 7.5, 150 mM NaCl). The reaction mixture was analyzed by SDS-PAGE with Coomassie staining or western blotting with antibodies against GST (cell signaling technology), His (cell signaling technology), ADPR (pan-ADP ribose, Millipore), Ub (abcam/ cell signaling technology). To detect PR-Ub, samples were phosphor-stained with Pro-Q diamond phosphostaining solution (Thermo Fisher). For Ubiquitin species quantification, a combined deconvoluted spectrum for the complete elution time of ubiquitin species was created with QualBrowser Xtract. Spectral intensities for Ubiquitin, ADPR-Ub and PR-UB species were summed up and displayed as a fraction of total Ubiquitin intensity. The prevalent species had in all cases a fraction of more than 99.5%.

#### Binding kinetics determination by biolayer interferometry (BLI)

Binding kinetics were determined with OctetRed system (Fortebio). His-Ub (12.5 μg/mL), His-Ub-ADPR (12.5 μg/mL), His-Rtn4 peptide-PR-Ub (12.5 μg/mL), His-Rtn4 peptide (50 μg/mL) were loaded onto Ni-NTA biosensor and equilibrated with binding buffer for the baseline. To examine the association rate, equilibrated sensors were transferred into solutions containing various concentration of GST-DupA H67A or GST-SdeA PDE H277A (0.15 - 10 μM, 1.5 - 100 μM, respectively). Dissociation of PDEs were initiated by placing sensor into in reaction buffer again. Association rate constant (kon), dissociation rate constant (koff), dissociation constant (Kd) values were calculated by Octet Data analysis software (ForteBio).

#### NMR titration assay

All NMR experiments were performed at 298 K on Bruker Avance spectrometer operating at proton frequencies of 800, 900, and 950 MHz. Titration experiments were performed with a 0.02 mM ^15^N-labeled Ub and ADPR-Ub protein samples (in standard 5mm NMR tube; all proteins were equilibrated with buffer containing in 20mM Tris pH 7.5, 20mM NaCl, 1mM TCEP) to which the non-labeled DupA protein were added stepwise until 3.2 and 4.0 times excess, respectively. For each titration step, BEST-[^15^N,^1^H]-TROSY and [^15^N,^1^H]-SOFAST-HMQC spectra were recorded to observe chemical shift perturbations (CSP) in comparison with the free Ub and ADPR-Ub spectra used as reference.

#### Initial structure preparation for MD simulation

For, Rtn4 peptide, the peptide DPTPVTSTVPAPT was constructed using tleap in the AmberTools14 program package[amber14], solvated with TIP3P (Jorgensen et al., 1983) and a neutralizing sodium ion in a periodic, truncated octahedral box, and simulated with the Amber FF14SB force field (Maier et al., 2015). The system was energy minimized using the sander module of Amber16[amber16], followed by molecular dynamics equilibration (5 ns) and production runs (100 ns) using particle-mesh Ewald electrostatics, a 10-Å cut-off for non-bonded real-space interactions, SHAKE constraints (Ryckaert et al., 1977) for bonds containing hydrogen atoms, Langevin dynamics (γ = 1 ps^-1^) at 300 K, and a Berendsen barostat (Berendsen et al., 1984) (τ = 2 ps) with isotropic position scaling to 1 atm, executed with the pmemd.cuda engine in Amber16.

For DupA with Ub-PR-Sub in DupA position, the initial structure was modeled according to the complex of DupA with Ub. The adenosine monophosphate was removed, obtaining an Ub-PR fragment. As peptide structure (Sub), a sterically suitable structure from the peptide production run was placed into the active side of DupA and covalently attached to the oxygen of the phosphoribose. Small steric clashes were manually removed.

For DupA with Ub-PR-Sub in SidE position, the initial structure was generated by aligning the structure of DupA with Ub-PR-Sub bound in DupA position to SidE PDE:Ub complex (PDB: 5ZQ3 (Wang et al., 2018)). The alignment was performed using the Multiseq tool (Roberts et al., 2006) in VMD 1.9.2 (Humphrey et al., 1996). Coordinates of Ub and DupA were saved. Ub-PR-Sub was aligned onto the DupA-Ub structure. Ub-PR-Sub and DupA coordinates were saved, combined, and steric clashes manually removed.

For SdeA PDE with Ub-PR-Sub in DupA position, the initial structure was generated based on the SdeA PDE structure (2.8 Å resolution, PDB: 6G0C (Kalayil et al., 2018)). Unresolved regions were modeled with the program MODELER (Webb and Sali, 2017).The modeled SdeA PDE structure was aligned to the DupA structure with Ub-PR-Sub in DupA position using the Multiseq tool in VMD 1.9.2. Coordinates of Ub-PR-Sub and SdeA PDE were saved, combined, and steric clashes manually removed.

#### SdeA PDE with Ub-PR-Sub in SidE position

The previously modeled SdeA PDE structure with Ub-PR-Sub in DupA position was aligned onto SidE PDE Ub (as described previously). The coordinates of Ub and SdeA PDE were saved. Ub-PR-Sub was aligned onto the SdeA PDE Ub structure. The coordinates of Ub-PR-Sub and SdeA PDE were saved, combined, and steric clashes removed.

#### Force field construction for ARG-PR-SER linker

Atom types were assigned for ARG according to the protein force field Amber FF14SB (Maier et al., 2015), for SER according to phosphoserine with single protonated phosphate group (Homeyer et al., 2006), SER-PO2(OH)), provided at http://research.bmh.manchester.ac.uk/bryce/amber), and for PR according to the RNA force field ParmBSC0 (Pérez et al., 2007) including the Χ_OL3_ correction (Banáš et al., 2010, Zgarbová et al., 2011)·

The initial ARG-PR-SER structure was based on the DupA structure with a bound ADP-ribosylated Ub (Ub-ADPR). The adenosine monophosphate was removed, obtaining an Ub-PR fragment. The ARG from Ub was truncated between the Cγ and Cδ position, i.e, a methylated guanidine bound to the C1’ of the phosphoribose. The phosphate at the other side was methylated, mimicking the Cβ of serine. The partial charges of the remaining neutral fragment were determined as follows: The geometry was energy minimized and restraint electrostatic potential (R.E.S.P) (Bayly et al., 1993) charges were calculated using the R.E.D. program (Dupradeau et al., 2010) (v. III.52) with Gaussian09[g09D] (RESP-A1: HF/6-31G^∗^ Connolly surface algorithm, 2 stage RESP fit qwt = 0.0005/.001, charge value accuracy ± 1.10^−4^ e). One hydrogen of each capping methyl group was removed, where PR was connected to ARG or SER, respectively. The original ARG (backbone and side chain up till Cγ) and SER (backbone) had a total net charge of 0.001124 e. Therefore, the partial charges of the new parameterized fragment were adjusted to maintain a net charge of −0.001124 e. The combination of original and new partial charges resulted in a neutral ARG-PR-SER fragment.

Parameters for the bond, angles, and dihedrals between the ribose and the phosphate were taken from the RNA force field. The parameters for the connection between phosphate and serine were taken from the phosphoserine force field with atom types renamed for the phosphate according to the RNA force field. The bond and angle parameters between the C1’ position of the ribose and the arginine were taken from the RNA force field and the atom types were adapted accordingly. Some dihedral angles in the RNA force field are defined via the C8 atom (adenine). As there are no corresponding atoms in the present system, we assigned the dihedrals to Cζ (ARG) according to 180°-C8 value.

#### Molecular dynamic simulation

All MD simulations were run with Amber16. Systems were prepared with the tleap program from AmberTools14. Proteins were set into a periodic, truncated octahedral box and solvated with TIP3P water (Maier et al., 2015), with a minimum 15-Å water layer to the box edges. The systems were neutralized and additional NaCl was added to mimic a salt concentration of 150 mM. Proteins and peptides were described with the Amber FF14SB force field (Maier et al., 2015). The force field parameters for the ARG-PR-SER linker are described in the preceding paragraph. HIS407 in SdeA PDE and HIS189 in DupA were doubly protonated. Other amino acid protonation states were assigned according to the PROPKA3.0 as part of the PDB2PQR web server (Vinet and Zhedanov, 2011) (pH = 7, http://nbcr-222.ucsd.edu/pdb2pqr_2.0.0/). Energy minimization was followed by equilibration (5 ns) and production runs (5 μs per system) using the same simulation settings as for the free substrate peptide.

#### Identification of novel substrates of SdeA by DupA trapping mutant

DupA eluted proteins were denatured by addition of 1 volume of 8 M urea in 50 mM Tris pH 8, cysteines were reduced and alkylated with TCEP and chloroacetamide. Proteolytic digest was performed for 3 h with 0.5 μg LysC (Wako) and after dilution to < 2 M Urea with 0.5 μg Trypsin (Promega) over night at 37°C. Tryptic peptides were desalted by Stage-Tips and analyzed on a Q Exactive HF (Thermo Fisher) coupled to an easy-LC 1200 (Thermo). In brief, peptides were separated with a non-linear 70 min gradient from 5%–35% solution B (80% Acetonitrile, 0.1% formic acid) on a 20 cm column packed with 1.9 μm C18 material (Dr. Maisch) and injected online into the mass-spectrometer. Survey scans were recorded with a resolution of 60,000 and the 15 most abundant precursor ions were subjected to HCD fragmentation. Data analysis was performed with MaxQuant 1.6.11 against the uniport human reference proteome database (December 2017) and the *Legionella pneumophila* reference proteome (December 2017). Label-free quantification was performed by MaxLFQ quantification with activated match-between-runs. Statistical testing and GO annotation were done with Perseus (1.6.1.1). Missing values in the control samples (Not infected and ΔSidE) were amputated and proteins, that were significantly enriched in WT and/or ΔDupA2 samples were identified by 5% FDR corrected T-Tests. Geneontology (GO) terms that were overrepresented among the potential SidE substrates were detected by a FDR 5% corrected test by the Panther website (Mi et al., 2019). Redundant terms were removed by Revigo (Supek et al., 2011) and the enrichment factors of the top hits were plotted with GraphPad Prism.

#### Identification of PR ubiquitination serine site on TEX264

GFP-Tex264 was enriched by non-denaturing IP from Hek293T cell and PR-ubiquitinated on beads with SdeA. SdeA and free Ubiquitin was removed by stringent washing with 8 M Urea. PR-Ubiquitinated Tex264 was digested on beads in 1 M Urea with Trypsin Gold (Promega) for 1 h and subsequently desalted by C18 StageTips and analyzed on QExactive HF (ThermoFisher) coupled to an easynLC 1200 (ThermoFisher) by data-dependent HCD fragmentation of precusors with charge state 4-7. Data-analysis was done with StavroX 3.6.6.6 with PR-Ubiquitin set as cross-linker.

#### Western Blotting and Immunoprecipitation

Tris-Glycine gels were used for SDS-PAGE followed by western blotting. Quantification of western blots was done using Image Lab software of Bio-rad. At least 3 independent experiments were performed and band intensities were normalized to loading control. p values were determined using Student’s t test. For immunoprecipitation, cells were lysed in immunoprecipitation buffer (50 mM Tris-Hcl, pH 7.5, 150 mM NaCl, 1% Triton X-100, 1 mM PMSF, protease inhibitor cocktail (Sigma Aldrich)), mixed with 10ul agarose conjugated Myc beads (SantaCruz Biotechnology), and incubated for 4 h at 4°C with end to end rotation. Beads were washed twice in IP buffer containing 400 mM NaCl. Proteins were eluted by boiling with 2X gel loading dye followed by western blotting.

#### Confocal imaging and image analysis

Confocal imaging was done using the Zeiss LSM780 microscope system. Ar-ion laser (for GFP excitation, CellROX Green, Alexa Fluor 488 with the 488 nm line), a HeNe laser (for mCherry, Alexa Fluor 546 with the 543 line) and a HeNe laser (for Alexa Fluor 633 and mitoTracker Deep Red with the 633 line) were used with 63 × 1.4 NA oil immersion objective. Images were analyzed in FIJI. Briefly images were converted to 8 bit, binary, thresholded, skeletonized for analysis of ER morphology. 50um^2^ ROIs near the cell periphery were used to quantify three way junctions containing Lnp1.Atleast 40 cells taken from three independent experiments were used for all analysis.

#### STED imaging

STED images of *Legionella* in HA-FAM134B expressing cells were acquired on a Leica TCS SP8 STED 3X using a 93 × 1,3 Glyc motCORR STED white objective lens. Fluorescence signal of Star 580 and Star 635P was excited at 580 and 650 nm and detected at 590 – 610 nm and 660 to 740 nm, respectively using Hybrid Detectors (HyD) in photon counting mode. Images were recorded with a pixel size of 27 nm, a dwell time of 1,5 μs and 10 line accumulations. For 2D STED imaging a pulsed 775 nm laser was used and time gated detection from 0,5 – 6,0 ns was applied. Data were post processed using the Leica’s Lighting image information extraction software. The image is a Maximum Intensity projection of 9 optical sections.

### Data and Code Availability

The atomic models of crystal structures reported in this paper have been deposited in Protein Data Bank (PDB: 6RYA and 6RYB). Original, unprocessed data from this manuscript have been deposited to Mendeley Data at: https://doi.org/10.17632/bkwjctz23n.1

## Supplemental Information

Document S1. Figures S1–S8 and Table S1

Table S2. List of PR-Ubiquitinated Substrates from Cells Infected with *Legionella* Strains, Related to Figure 4 Mass spectrometry results showing consistently significantly enriched proteins from *Legionella* infection.

Video S1. Molecular Dynamics Simulation of PR-Ubiquitinated Rtn4 Located in SidE Ub Position with DupA, Related to Figures 3 and S4 Ub moiety from PR-ubiquitinated Rtn4 is superimposed on Ub from SidE Ub complex structure and subjected to MD simulation for 5 μs.

Video S2. Molecular Dynamics Simulation of PR-Ubiquitinated Rtn4 Located in SidE Ub Position with SdeA PDE, Related to Figures 3 and S4 Ub moiety from PR-ubiquitinated Rtn4 is superimposed on Ub from DupA:Ub complex structure and subjected to MD simulation for 5 μs.

Video S3. Molecular Dynamics Simulation of PR-Ubiquitinated Rtn4 Located in DupA Ub Position with DupA, Related to Figures 3 and S4 Ub moiety from PR-ubiquitinated Rtn4 is superimposed on DupA:Ub complex structure and subjected to MD simulation for 5 μs.

Video S4. Molecular Dynamics Simulation of PR-Ubiquitinated Rtn4 Located in DupA Ub Position with SdeA PDE, Related to Figures 3 and S4 Ub moiety from PR-ubiquitinated Rtn4 is superimposed on DupA:Ub complex structure and subjected to MD simulation for 5 μs.

Document S2. Article plus Supplemental Information
