# Supplementary material for: Regulation of Phosphoribosyl-Linked Serine Ubiquitination by Deubiquitinases DupA and DupB
Source: Mol Cell. 2020 Jan 2;77(1):164–179.e6. doi: 10.1016/j.molcel.2019.10.019 (PMC6941232; doi:10.1016/j.molcel.2019.10.019)
Supplement: Document S1. Figures S1–S8 and Table S1 [file mmc1.pdf]

Molecular Cell, Volume *77*

Supplemental Information

Regulation of Phosphoribosyl-Linked Serine Ubiquitination by Deubiquitinases DupA and DupB

Donghyuk Shin, Rukmini Mukherjee, Yaobin Liu, Alexis Gonzalez, Florian Bonn, Yan Liu, Vladimir V. Rogov, Marcel Heinz, Alexandra Stolz, Gerhard Hummer, Volker Dötsch, Zhao-Qing Luo, Sagar Bhogaraju, and Ivan Dikic

Supplemental Information

Regulation of phosphoribosyl-linked serine ubiquitination by deubiquitinases DupA and DupB

Shin et al.

Supplemental figure S1

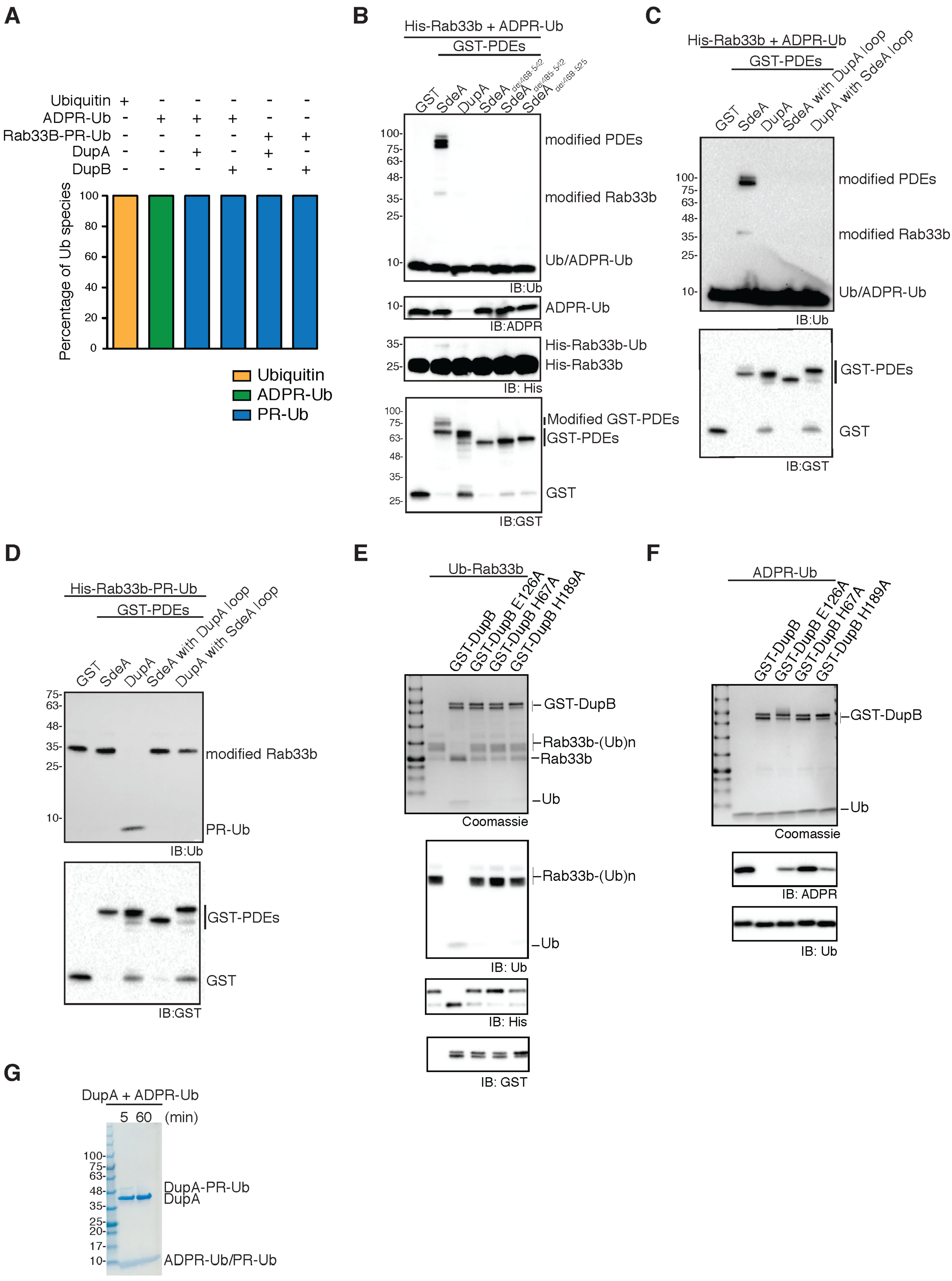

Supplemental figure S1. Control data related to Figure 1 and Figure 2. (A) PR-Deubiquitination assay of legionella lysates. PR-ubiquitinated-Rab33b was incubated with *Legionella* lysates as indicated. **(B and C)** PR-Ubiquitination assays of SdeA PDE loop deletion mutant, loop swapping mutant, respectively. **(D)** PR-Deubiquitination assay of DupA, SdeA PDE loop swapping mutant. **(E and F)** Deubiquitination assay of DupB wild-type and catalytically inactive mutants on PR-ubiquitinated Rab33b and ADPR-ubiquitin, respectively. **(G)** Time-course auto-PR-ubiquitination assay of DupA wild-type.

Supplemental figure S2

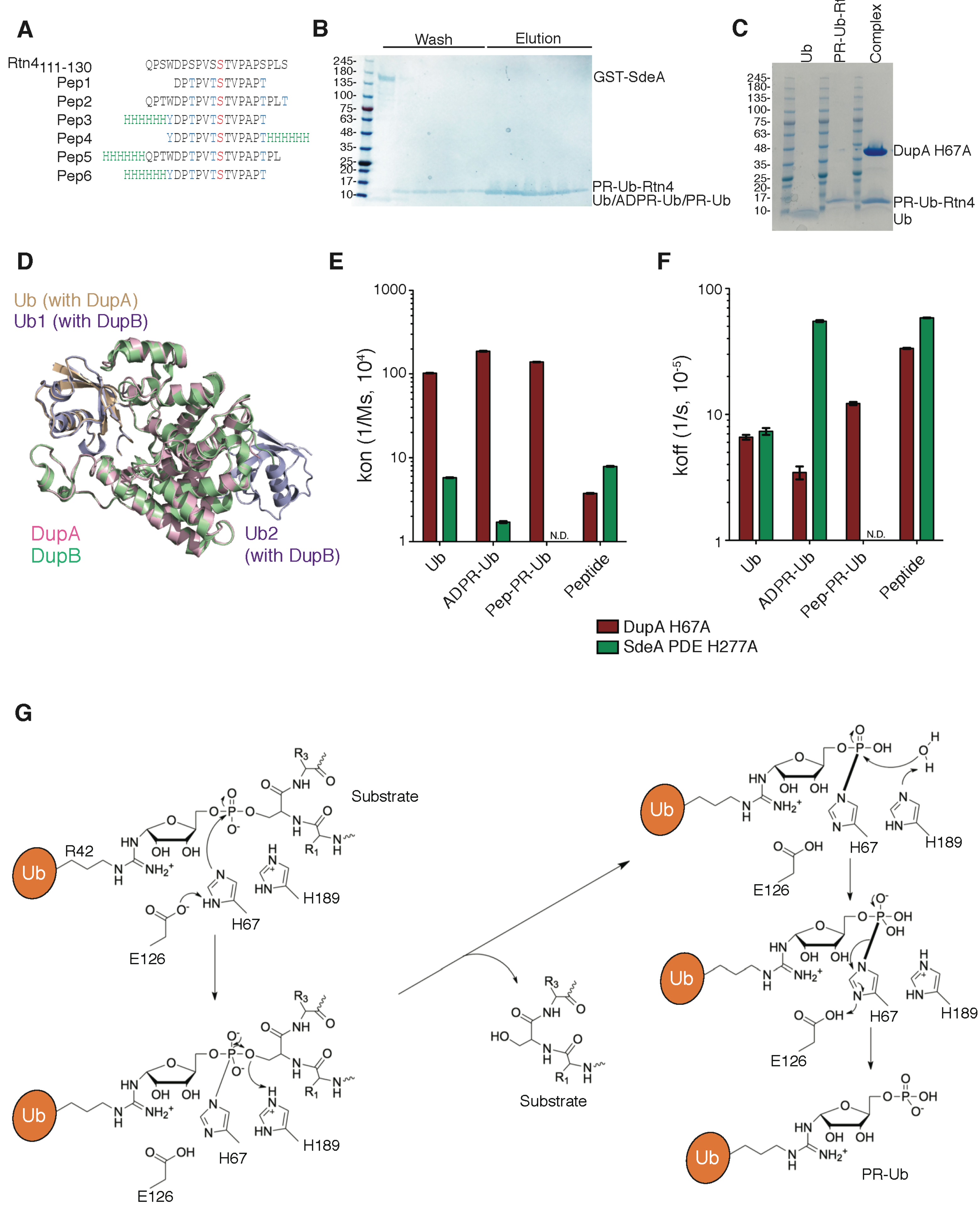

Supplemental figure S2. Synthesis of PR-ubiquitinated Rtn4 peptide and binding kinetics of $\text{DupA}_{\text{H67A}}$ and SdeA $\text{PDE}_{\text{H277A}}$ - related to Figure 3. (A) Original Rtn4 peptide was modified to have single serine residue for homogenous modification. **(B)** Purification of PR-ubiquitinated His-tagged Rtn4 peptide. **(C)** DupA H67A and PR-ubiquitinated Rtn4 complex mixture used for crystallization. **(D)** Superimposition of DupA:Ubiquitin complex structure with DupB (SdeD):Ubiquitin structure (PDB ID: 6B7P). **(E and F)** Association rate (k_{on}) and dissociation rate (k_{off}) of catalytically inactive $\text{DupA}_{\text{H67A}}$ and SdeA $\text{PDE}_{\text{H277A}}$ to ubiquitin species or peptide as indicated. Data are represented as mean ± S.E.M. *S.E.M; standard error of mean. **(G)** Proposed catalytic mechanism of PR-ubiquitin cleavage by DUPs.

Supplemental figure S3

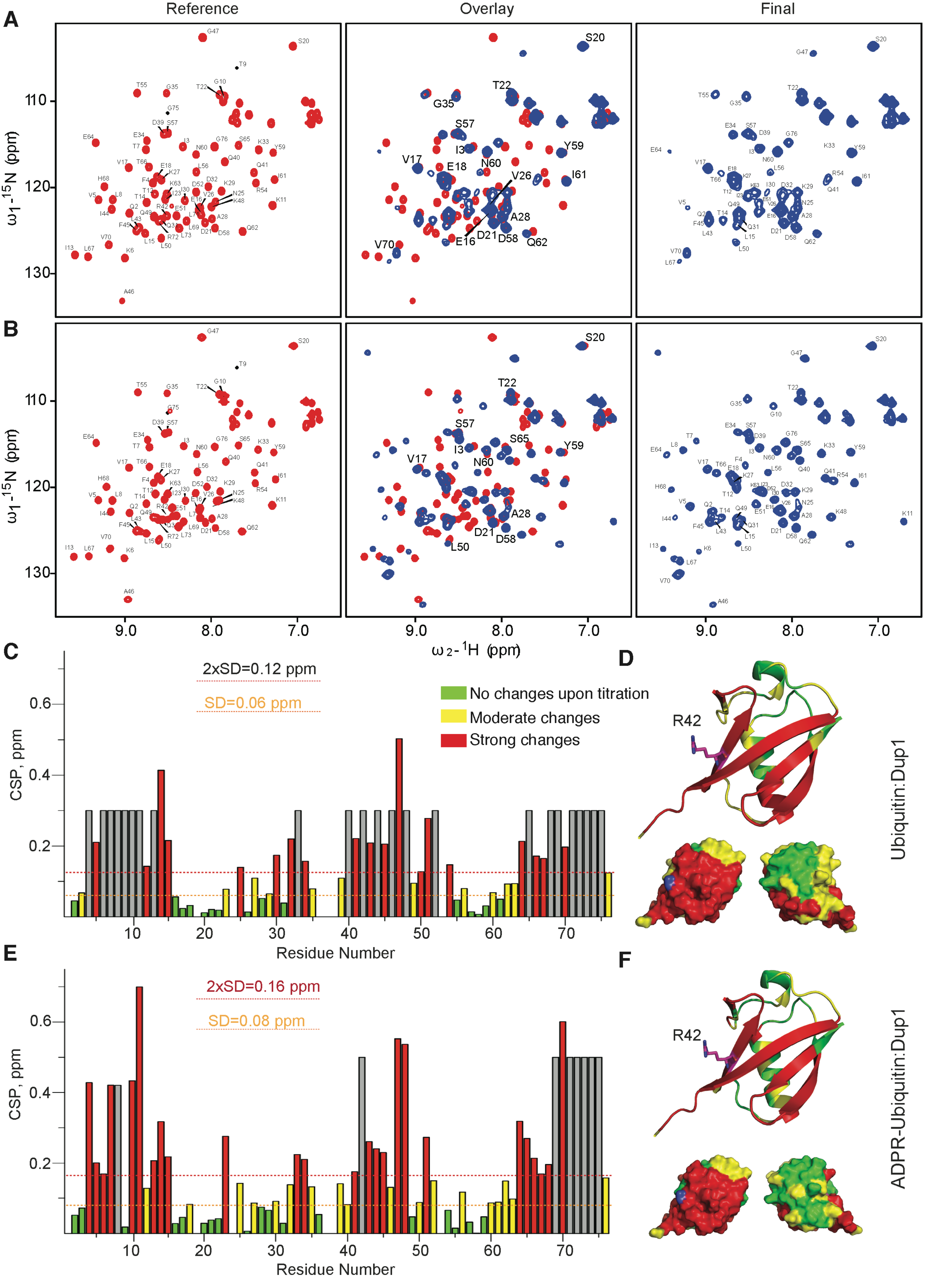

Supplemental figure S3. NMR titrations of the DupA$_{H67A}$ into ^{15}N-labeled Ub and ADPR-Ub - related to Figure 3. (A) Representative sections of [^{15}N,^{1}H]-SOFAST-HMQC spectra for free ^{15}N-labeled Ub (left), the Ub in presence of DupA (right), and overlay of both (middle). **(B)** The same experiments for 15N-labeled ADPR-Ub. Resonance assignments are shown where possible; the assignments for the Ub resonances at the final titration stage represent the most likely assignments based on the resonances of free Ub. While the Ub-DupA complex shows intermediate exchange dynamics with many resonances disappearing, the ADPR-Ub-DupA complex shows slow exchange. **(C)** CSP values observed for the ^{15}N-labeled Ub resonances upon titration with non-labelled DupA (at a molar ratio of 1:3.2) and mapped on the Ub protein sequence. Yellow and red lines indicate the 1x and 2x standard deviations (SD) calculated from CSP values of all Ub HN resonances which can be traced over the titration experiment. CSP bars for residues with small (CSP < SD), intermediate (SD < CSP < 2xSD) or strong (2xSD < CSP) CSPs are marked in green, yellow and red, respectively. CSP bars for residues with disappearing resonances are set to 0.3 ppm and colored gray. **(D)** CSP values mapped on the Ub protein structure (PDB ID: 1UBQ) shown as a ribbon diagram (upper panel) and as protein surfaces (lower panel, showing two orientations differing by a 180° rotation around the Y axis). Residues with small (CSP < SD), intermediate (SD < CSP < 2xSD) or strong (2xSD < CSP) CSPs were marked in green, yellow and red, respectively. All residues with disappeared HN signal were also marked red. The R42 sidechain is shown as sticks in the ribbon diagram and marked blue on the surface representations. **(E)** CSP values observed for the ^{15}N-labeled ADPR-Ub resonances upon titration with non-labelled DupA (at a molar ratio of 1:2) and mapped on the Ub protein sequence. Yellow and red lines indicate the 1x and 2x standard deviations (SD) calculated from CSP values of all Ub HN resonances which can be traced over the titration experiment. CSP bars for residues with small

(CSP < SD), intermediate (SD < CSP < 2xSD) or strong (2xSD < CSP) CSPs are marked in green, yellow and red, respectively. CSP bars for residues which could not be assigned due to unambiguity are set to 0.4 ppm and colored gray. **(F)** CSP values mapped on the Ub protein structure (PDB ID 1UBQ) shown as a ribbon diagram (upper panel) and as protein surfaces (lower panel, showing two orientations differing by a 180° rotation around the Y axis). Residues with small (CSP < SD), intermediate (SD < CSP < 2xSD) or strong (2xSD < CSP) CSPs were marked in green, yellow and red, respectively. All residues with disappeared HN signal were also marked red. The R42 sidechain is shown as sticks in the ribbon diagram and marked blue on the surface representations.

Supplemental figure S4

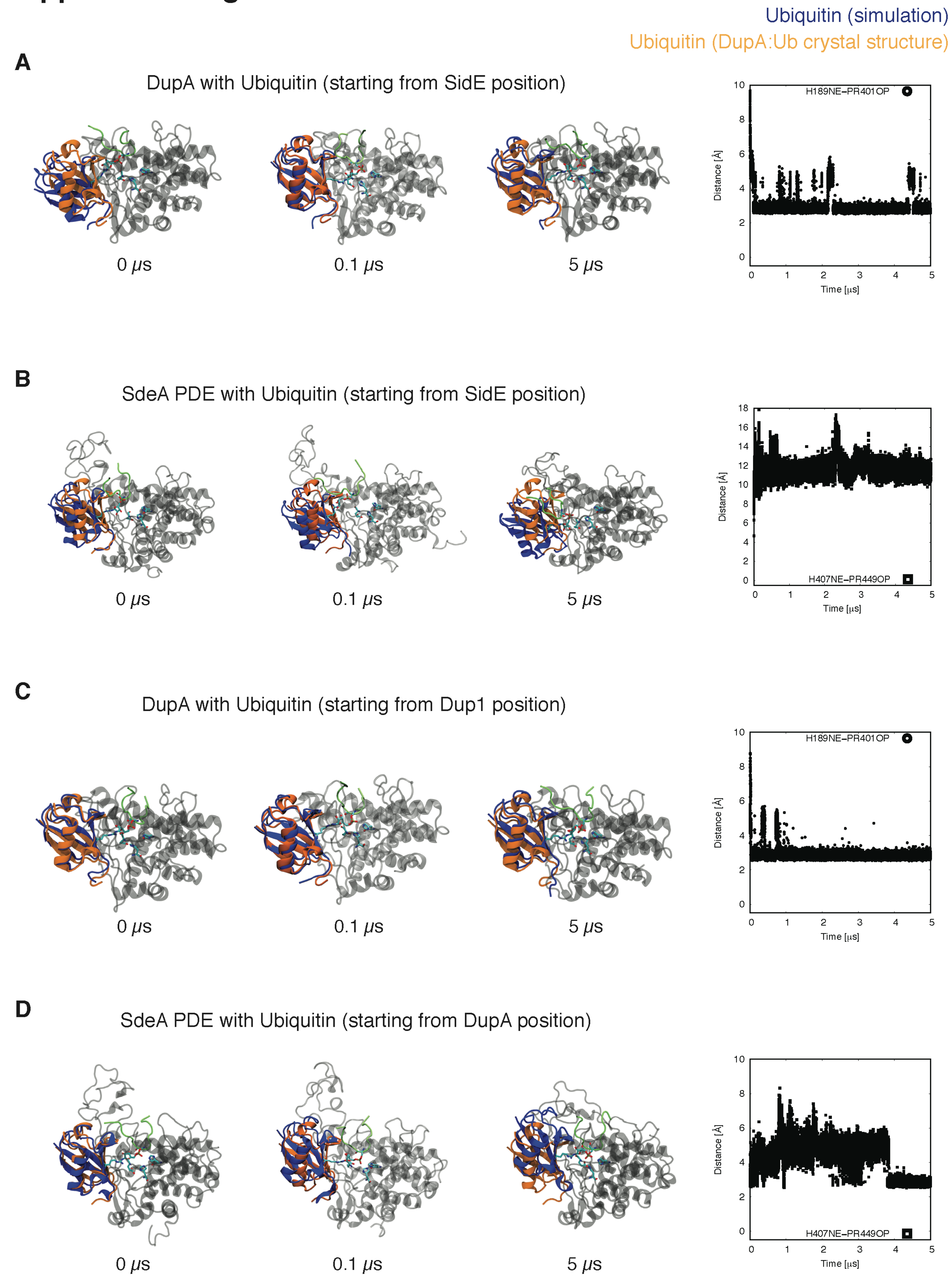

Supplemental figure S4. Molecular dynamics simulation of DupA and SdeA PDE with PR-ubiquitinated Rtn4 peptide - related to Figure 3. (A and B) MD simulation of PR-ubiquitinated-Rtn4 located in SidE ubiquitin position with DupA and SdeA PDE, respectively. **(C and D)** MD simulation of PR-ubiquitinated-Rtn4 located in DupA ubiquitin position with DupA and SdeA PDE, respectively. Subset graph shows distance between H189 of DupA or H407 of SdeA PDE and phosphate from PR-ubiquitinated Rtn4 peptide.

Supplemental figure S5

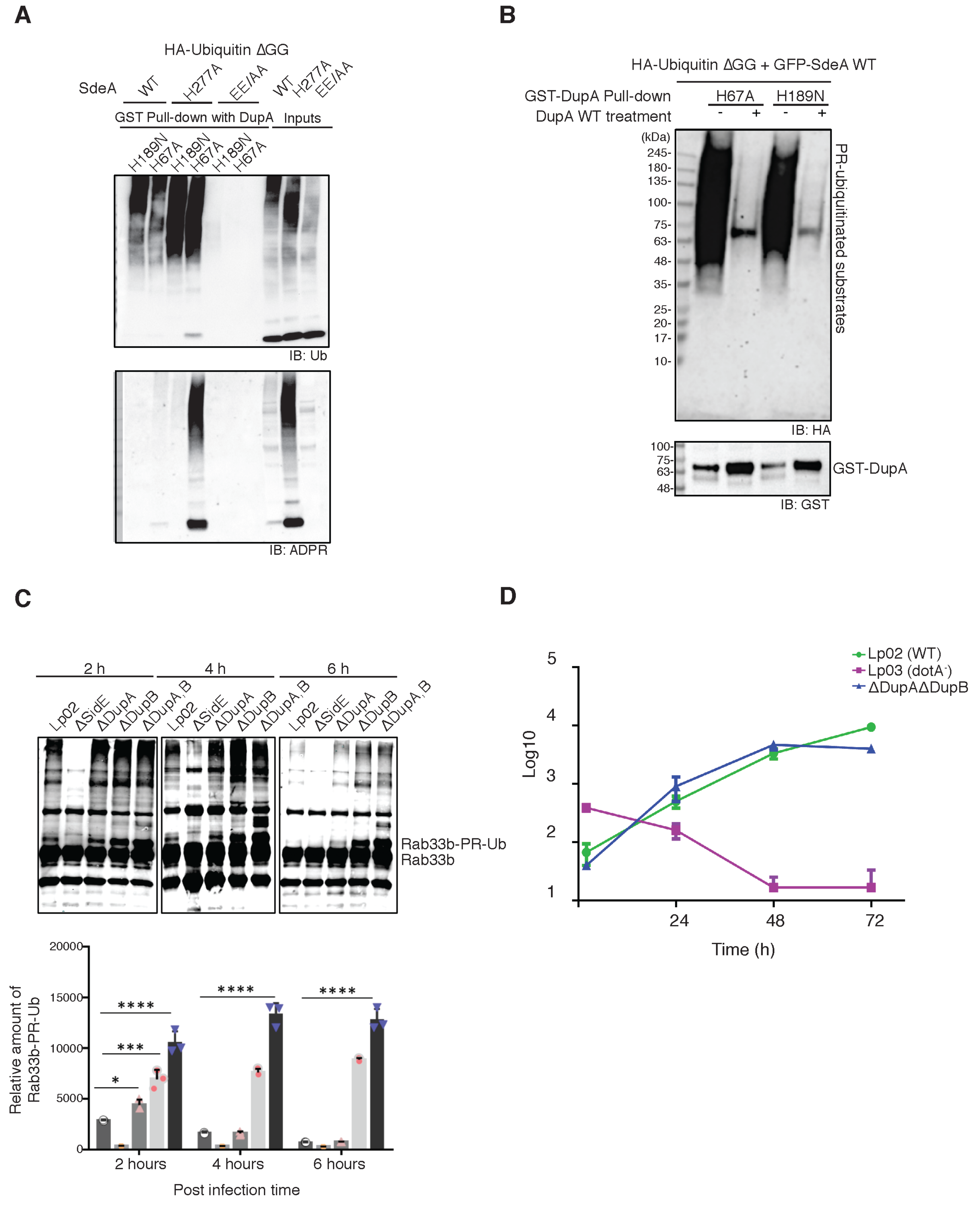

Supplemental figure S5. Enrichment of PR-ubiquitinated substrates from cells infected with Legionella strains - related to Figure 4. (A) Control Ubiquitin and ADPR blot for Figure 4A **(B)** Trapping endogenous PR-ubiquitinated substrates from cells transfected with SdeA and Ubiquitin *Δ*GG. Samples were further incubated with DupA WT to elute substrates. **(C)** PR-ubiquitinated Rab33b from cells infected with Legionella strains was monitored as indicated. **(D)** Mouse bone marrow-derived macrophages were infected with Legionella strains and the proliferation of the bacteria was monitored at the indicated time points. Data are represented as mean ± SD.

Supplemental figure S6

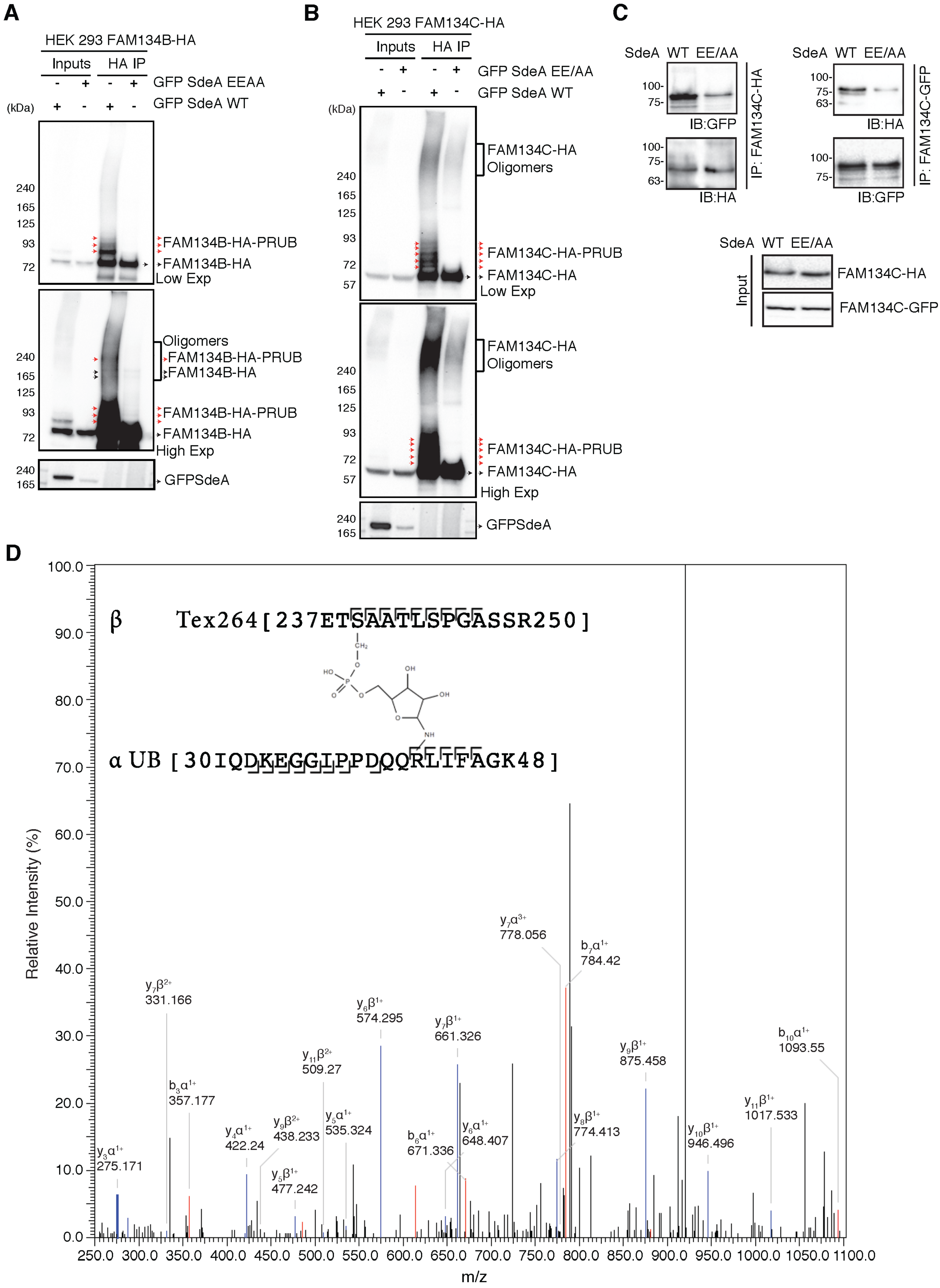

Supplemental figure S6-control data related to Figure 5 (A, B) HEK 293 HA-FAM134B or HA-FAM134C cells were transiently transfected with SdeA WT or EE/AA mutant, respectively. Soluble extracts were subjected to HA-IP and blotted against HA. Increased levels of the high molecular mass forms of HA FAM134B/C are immunodetected in SdeA WT transfected cells. **(C)** Lysates from cells co-transfected with HA-FAM134C, GFP-FAM134C and SdeA or SdeA(EE/AA) were used to immunoprecipitate HA-FAM134C and GFP-FAM134C. Oligomer formation was assessed by immunoblotting with GFP and HA respectively. **(D)** In vitro ubiquitinated Tex264 was digested with Trypsin and analyzed by LC-MS. PR-Ubiquitination could be mapped to Serine 239.

Supplemental figure S7

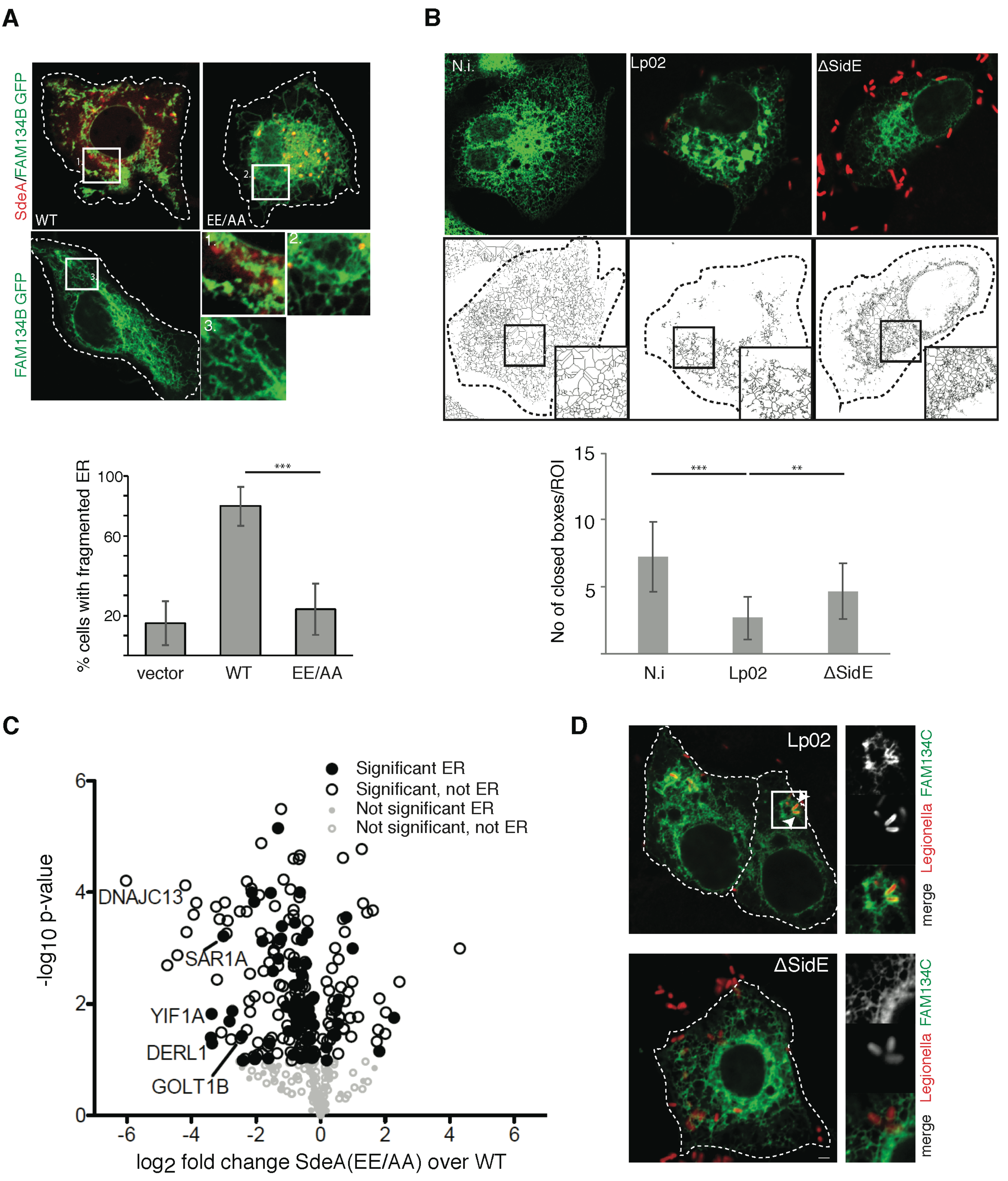

Supplemental figure S7-control data related to Figure 6

(A) Fragmentation of FAM134B marked ER in cells expressing SdeA. Graph represents 30 cells taken from 3 independent experiments. Error bars, represent standard deviation., $p<0.05$. **(B)** Confocal images taken after 2h of infection were processed with ImageJ (converted to binary and skeletonized). More interconnected ER tubules are seen for control and delSidE sets compared to cells with wild type Legionella. Error bars, represent standard deviation (C) Lysates from Hek293T cells expressing Lnp1-GFP, HA-SdeA or SdeA(EE/AA) were used for immunoprecipitation with GFP beads followed by mass spectrometric analysis to identify interactors of Lnp1 in both sets. **(D)** A549 cells were transfected with FAM134C-HA followed by infection with bacteria. 2 hours post infection, cells were fixed and immuno-stained using antibodies against *Legionella* and HA. Graph represents 30 cells taken from 3 independent experiments. Error bars, represent standard deviation, $p<0.05$

Supplemental figure S8

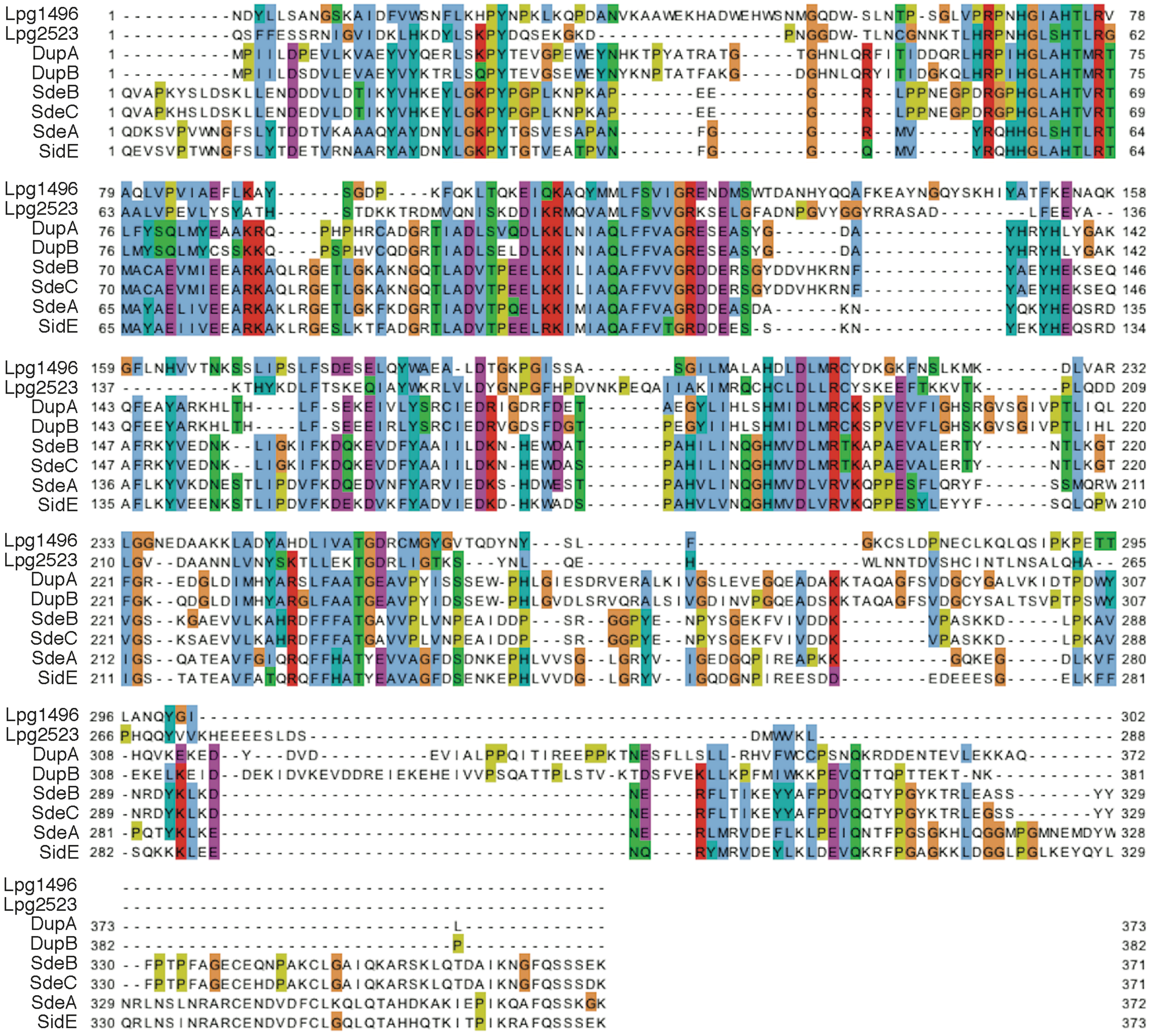

Supplemental figure S8. Multiple sequence alignment of Legionella PDEs related to figure 1. Sequences of eight PDE domains identified from pattern search were aligned using Clustal Omega (https://www.ebi.ac.uk/Tools/msa/clustalo/).

Table S1 |Data collection and refinement statistics – related to figure 2 and 3

	$DupA_{4-345}$	$DupA_{4-345}$:Ubiquitin
**Data Collection**		
Space group	P 61 2 2	C 1 2 1
Cell dimensions		
a, b, c (Å)	87.259 87.259 612.618	116.33 ,67.14, 182.75
α, β, γ (°)	90, 90, 120	90, 90, 90
R_{merge}	0.1309 (2.371)	0.095 (0.484)
R_{pim}	0.02865 (0.5073)	0.065 (0.351)
$CC_{1/2}$	0.999 (0.452)	0.995 (0.672)
CC^*	1 (0.789)	0.999 (0.897)
I / σI	19.11 (1.24)	7.28 (1.39)
Completeness	99.88 (99.22)	99.86 (99.96)
Redundancy	21.7 (22.1)	2.6 (2.4)
**Refinement**		
Resolution (Å)	47.59 – 2.315 (2.398 -2.315)	49.07 – 2.21 (2.289 – 2.21)
No. reflections	62183 (6017)	65182 (7030)
R_{work}/R_{free}	0.2174 /0.2475	0.2088 / 0.2888
No. atoms	7659	9240
Macromolecules	7492	9240
Solvent	167	0
B-factors		
Macromolecules	63.10	46.95
Solvent	54.65	-
R.m.s deviations		
Bond lengths (Å)	0.008	0.016
Bond angles (°)	0.94	2.13
